# Protocol for the process evaluation of the GOAL trial: investigating how comprehensive geriatric assessment (CGA) improves patient-centred goal attainment in older adults with chronic kidney disease in the outpatient setting

**DOI:** 10.1136/bmjopen-2023-076328

**Published:** 2024-08-01

**Authors:** Sarah Therese Fox, Ruth Hubbard, Andrea Valks, Misa Matsuyama, Emarene Kalaw, Andrea Viecelli, Eunise Martha Aquino, David Johnson, Monika Janda

**Affiliations:** 1 Centre for Health Services Research, The University of Queensland, Woolloongabba, Queensland, Australia; 2 Internal Medicine Services, The Prince Charles Hospital, Chermside, Queensland, Australia; 3 Department of Geriatric Medicine, Princess Alexandra Hospital, Woolloongabba, Queensland, Australia; 4 Australasian Kidney Trials Network, The University of Queensland, Brisbane, Queensland, Australia; 5 Department of Kidney and Transplant Services, Princess Alexandra Hospital, Woolloongabba, Queensland, Australia; 6 Centre for Kidney Disease Research, Translational Research Institute, Brisbane, Queensland, Australia

**Keywords:** Clinical Trial, Health Services for the Aged, Chronic renal failure, Patient Reported Outcome Measures, QUALITATIVE RESEARCH

## Abstract

**Introduction:**

The GOAL Cluster Randomised Controlled Trial (NCT04538157) is now underway, investigating the impact of comprehensive geriatric assessment (CGA) for frail older people with chronic kidney disease (CKD). The primary outcome is the attainment of patient-identified goals at 3 months, assessed using the goal attainment scaling process. The protocol requires a dedicated process evaluation that will occur alongside the main trial, to investigate issues of implementation, mechanisms of impact and contextual factors that may influence intervention success. This process evaluation will offer novel insights into how and why CGA might be beneficial for frail older adults with CKD and provide guidance when considering how to implement this complex intervention into clinical practice.

**Methods and analysis:**

This process evaluation protocol follows guidance from the Medical Research Council and published guidance specific for the evaluation of cluster-randomised trials. A mixed methodological approach will be taken using data collected as part of the main trial and data collected specifically for the process evaluation. Recruitment and process data will include site feasibility surveys, screening logs and site issues registers from all sites, and minutes of meetings with intervention and control sites. Redacted CGA letters will be analysed both descriptively and qualitatively. Approximately 60 semistructured interviews will be analysed with a qualitative approach using a reflexive thematic analysis, with both inductive and deductive approaches underpinned by an interpretivist perspective. Qualitative analyses will be reported according to the Consolidated criteria for Reporting Qualitative research guidelines. The Standards for Quality Improvement Reporting Excellence guidelines will also be followed.

**Ethics and dissemination:**

Ethics approval has been granted through Metro South Human Research Ethics Committee (HREC/2020/QMS/62883). Dissemination will occur through peer-reviewed journals and feedback to trial participants will be facilitated through the central coordinating centre.

**Trial registration number:**

NCT04538157.

**STRENGTHS AND LIMITATIONS OF THIS STUDY**

- This is a prespecified, theory-based process evaluation allowing both deductive and inductive analyses of this complex intervention.
- The approach is anchored in sound mixed methodology, reflecting both the updated Medical Research Council guidance, as well as other published guidance for process evaluations of cluster randomised controlled trials.
- The lead researcher is independent of the main trial team. Furthermore, the analyses will be conducted without knowledge of the main trial outcomes and in a deidentified format. These measures improve objectivity.
- Limitations include that the process evaluation plan was not used to inform the trial intervention design and development. It will not feedback on issues identified during the trial. While this reduces risk of bias and maintains objectivity, it limits the scope of the process evaluation to problem-solve implementation difficulties during the trial.

## Introduction

 As the global population ages, the impacts of frailty, multimorbidity and psychosocial vulnerability accumulate.^1 2^ Frail older adults require a holistic, patient-centred approach to care, with a focus on outcomes relevant to their personal circumstances.^3^ Comprehensive geriatric assessment (CGA) is an example of such care.^4^ It is a multidimensional, multidisciplinary assessment of medical, psychological and functional capabilities of frail older people that links to an integrated and coordinated care plan to improve function and patient-centred outcomes.^5^

Despite a number of randomised controlled trials (RCT) investigating the impact of CGA in different settings and patient populations, results have been mixed and it has been difficult to show definitive benefit in different contexts.^6^ For example, CGA applied in the inpatient setting shows clear benefit by increasing the likelihood of patients remaining alive and in their home at 12 months, whereas evidence of benefit is limited for CGA delivered in the outpatient or postdischarge setting.^6^,^8^ A recent meta-analysis of CGA in both the inpatient and outpatient settings showed mixed benefits, with no impact on length of stay but improvement in quality of life and caregiver burden.^9^

The reason for the differential efficacy of CGA in various settings has been difficult to tease apart with reference to RCT results alone. Comparison of trial analyses has been limited by significant heterogeneity not just in outcome measures, but also in the content and processes of the intervention itself.^7^ Furthermore, the details of the intervention are not always clear in the literature. The mixed results seen in the literature likely reflect that CGA is a complex healthcare intervention. Despite the broadly accepted general definition of CGA, the details of intervention characteristics vary significantly between trials. Variations are reflected in components and processes of the intervention, contributions and roles of various healthcare professionals, and different settings and patient groups.^5^ CGA is also heavily influenced by context and implementation factors, including but not limited to the people delivering the intervention, processes of change, leadership, and educational and data resources. This means that even if the processes of CGA were delivered uniformly, outcome effects would vary in different trial and clinical contexts. Therefore, it is not enough to know whether CGA might work in certain situations, as RCTs might indicate, but to understand how and why it can be effective in different situations. However, this is generally not possible with reference to the trial output data alone. Rather, it requires an analysis of implementation and process factors, thus supporting translation of trial results into effective policy and clinical practice.^10 11^

A few recent clinical trials of CGA have been supported by dedicated process evaluations, which have improved trial interpretation. For example, a trial of CGA for community-dwelling older adults concluded that participants expressed the need for a holistic view and that the proactive nature of the intervention delivered unexpected help.^12^ A trial of perioperative CGA for emergency abdominal surgery, in which CGA did not improve survival or length of stay, was strengthened by a dedicated process evaluation which showed that intervention fidelity in the trial was poor, social aspects of change were challenging and resources were poor.^13^ Another process evaluation of a behaviour change intervention for older adults highlighted the important of assessing the fidelity of a trail intervention. In that trial, the quality of motivational interviewing and goal-setting was poor, with 90% of goals set having a low potential for behaviour change, and only 1 of 11 motivational interviewing thresholds meeting a quality threshold.^14^ Questions remain about what are the key processes without which CGA lacks benefit, including the relative importance of goal-setting, care planning and follow-up. The role of patient and caregiver expectation in shaping outcomes is also poorly understood. Furthermore, there is doubt about how CGA might work in the outpatient setting when delivered primarily by a single clinician rather than a multidisciplinary team.

CGA may be particularly suited to older adults with chronic kidney disease (CKD) who have high degrees of frailty and functional impairment.^15 16^ Process evaluations have occurred for the implementation of specific CGA programmes in nephrology care. These have suggested CGA can help anticipate and manage risks, identify problems and focus decision-making to be patient-centred.^17 18^ However, a challenge for CGA in this cohort is ensuring that goals and assessments are communicated appropriately to patients. Effective multidisciplinary working has been identified as important in ensuring CGA is beneficial for this group.^19^

### The GOAL trial

The GOAL trial (*Comprehensive Geriatric Assessment for Frail Older People with Chronic Kidney Disease to Increase Attainment of Patient-Identified Goals - A Cluster Randomised Controlled Trial*) is a pragmatic, cluster RCT of CGA for frail older adults with CKD conducted in the outpatient setting. The pragmatic design of the trial means that geriatricians are asked to provide CGA as they usually would in their clinical practice. This is a comprehensive, multidimensional assessment provided by a geriatrician in the outpatient setting. No proforma or structure to the CGA is mandated, and it is at the discretion of the geriatrician to what degree they employ multidisciplinary input or ongoing follow-up. The primary outcome of interest is goal-attainment scaling, with secondary analyses focusing on quality of life as well as clinical and operational outcomes such as readmissions, return to home, mortality and cost-effectiveness. The protocol for the main GOAL trial is published.^20^ Recruitment for the GOAL trial concluded in July 2023. On completion of trial recruitment and follow-up, we will be conducting a dedicated process evaluation of the GOAL Trial, focusing on implementation, recruitment, reach, context and causal pathways, as described in this protocol.

The GOAL trial results are much anticipated and will improve our understanding of optimal models of care for people living with frailty and CKD. The trial design is deliberately pragmatic, to optimise the trustworthiness of the results when applied to the non-trial context. However, because the intervention, CGA, is so complex, the main trial data results will be difficult to interpret without the process evaluation work that sits alongside. The process evaluation will improve understanding of how resources, people, context and causal mechanisms work together in the context of the GOAL trial to augment outcomes. The process evaluation will also improve our understanding of how CGA is experienced by those patients with frailty and CKD who receive the intervention in the outpatient setting. When the GOAL results are available, the next question will be how the results can be translated to health service delivery in an optimal way. The process evaluation analysis will play an important role in closing the know-do gap that so often plagues complex interventions such as CGA.^21^

## Methods and analysis

### Broad aims and design

The aim of the process evaluation is to understand how outpatient CGA might be effective in improving health outcomes for frail older adults with CKD, in the context of a cluster RCT. Our approach incorporates the Medical Research Council (MRC) guidance for developing and evaluating complex interventions published in 2021, and for process evaluation of complex interventions, updated in 2015 from previous guidance published in 2008.^22^,^24^ Specifically, we aim to investigate implementation, mechanisms of impact and context of the CGA intervention. In addition to addressing these MRC domains, we will structure our study design and research questions according to published guidelines for process evaluations of cluster RCTs.^25^

A key objective is an exploration of how CGA was implemented in the trial context, including recruitment processes (who was recruited and how), and how the intervention was delivered to patients. Other key objectives include the exploration of patient response to the intervention and perceived acceptability to patients, unexpected consequences of the intervention and broader contextual factors that influenced implementation and patient experience. The aim is that this exploratory analysis will allow further refinement of programme theory, to understand the impact of contextual factors on causal mechanisms.

The design of the process evaluation incorporates four iterative stages: development of programme theory, generation of research questions, generation of data and analysis of data. The process evaluation will occur without knowledge of trial outcomes. The process evaluation includes key investigators involved in the main GOAL trial. However, the initial analysis will be conducted by one independent researcher, the first author, who is not involved in the main trial and will not feedback results while the trial is ongoing. Furthermore, collection of data specific to the process evaluation, such as semistructured interviews, will only commence once each site has completed the last-patient, last-visit to limit the process evaluation work influencing the main trial outcomes.

Data collection for the process evaluation will take place between August 2023 and June 2025. Analysis of process data will take place between June 2024 and August 2025.

### Overview of mixed methodology

**Figure 1:**
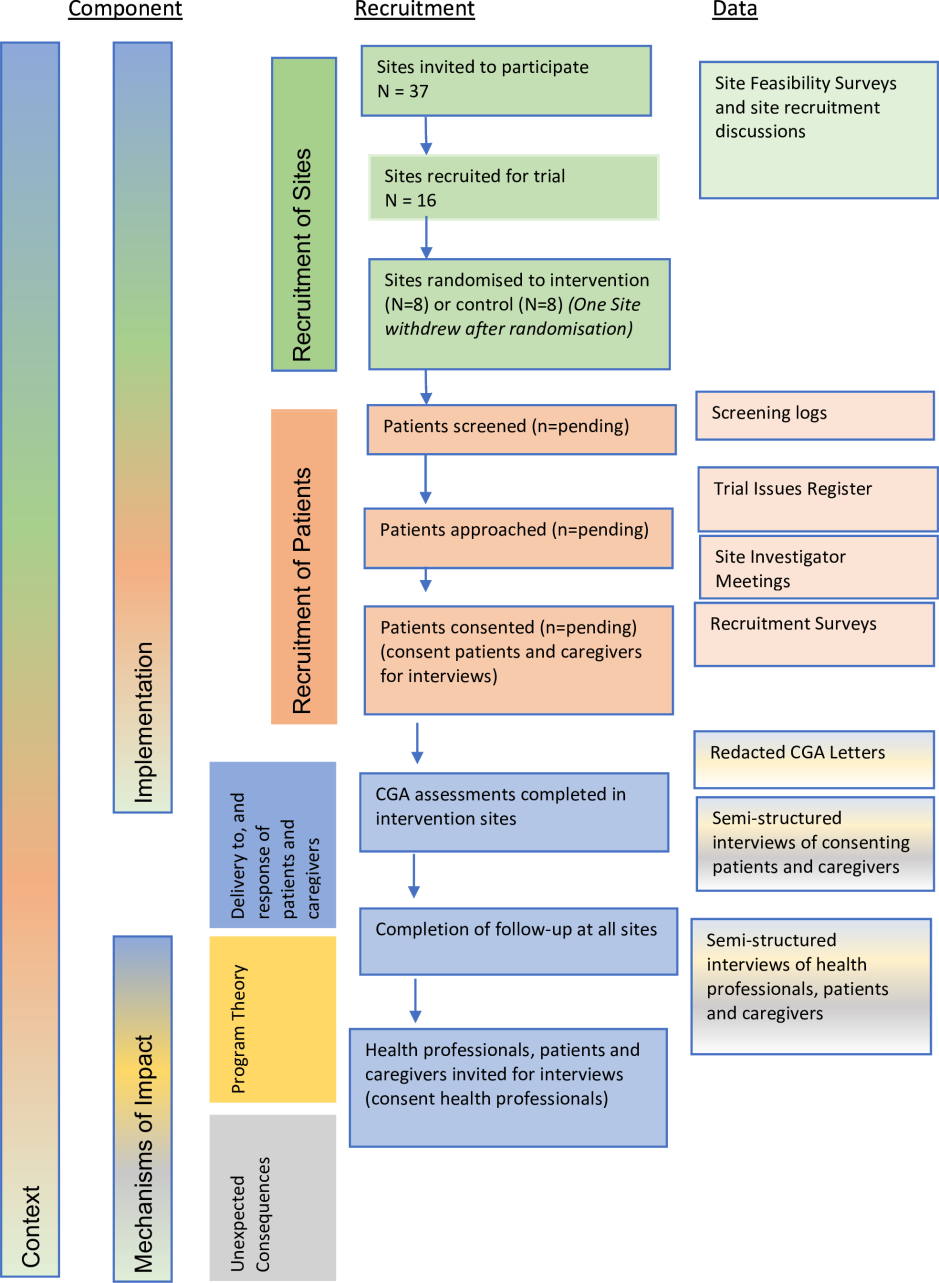
Recruitment for GOAL *(Comprehensive Geriatric Assessment for Frail Older People with Chronic Kidney Disease to Increase Attainment of Patient-Identified Goals - A Cluster Randomised Controlled Trial*) process evaluation. CGA, comprehensive geriatric assessment.

Data sources and recruitment processes for the process evaluation are presented in figure 1.

The process evaluation will allow for both deductive and inductive approaches, an iterative process to incorporate new learning through ongoing data collection, review and reflection. The process evaluation will predominantly be carried out by three researchers (STF, RH and MJ). One of these (STF) is not involved in the main outcomes trial and has no input into the main trial design, data collection or data analysis. The others (RH and MJ) are investigators in the main outcomes trial. Raw data collection will be carried out by STF. Analysis of qualitative data will be done by STF with contribution from MJ. The qualitative analyses will then be discussed with RH and other team members for the purposes of triangulation. A summary of the data collection methods that will be used to answer the relevant domains of the process evaluation are presented in figure 1 and table 1.

**Table 1:** Organisation of data collection method to address key questions in process evaluation for CGA in the GOAL (*Comprehensive Geriatric Assessment for Frail Older People with Chronic Kidney Disease to Increase Attainment of Patient-Identified Goals - A Cluster Randomised Controlled Trial*) trial

Component	Data source	Data collected
***Recruitment of clusters: who was recruited and how?*** *MRC domains: Implementation, acceptability, and barriers and enablers*	*Site meetings* ***Site recruitment surveys*** *Feasibility surveys*	What were the characteristics of clusters who agreed to participate and did this suggest any bias?How many of the approached sites (=clusters) agreed to participate?Why (or why not) did sites agree to participate? What were the barriers and enablers?How did COVID-19 impact the site’s participation?
***Response of clusters: how did clusters change or adapt due to the intervention?*** *MRC domains: Implementation, acceptability, and barriers and enablers*	*Issues registerSite meetingsOutcome data including where CGA was intended but not delivered* ***Recruitment surveysInterviews with health professionals***	Was there a local clinical trials nurse responsible for the trial?How did sites organise to provide CGA?To what extent was the CGA organised through an MDT?Was there evidence of effective clinical leadership?Were MDT Meetings present?What was the method of communication with the GP?How was communication organised between various stakeholders within sitesWhere was CGA provided on the sites? Was telehealth delivery used? Was this temporary for the trial?
***Recruitment of individuals: whichpatientswere recruited and how?*** *MRC domains: Implementation, acceptability, and barriers and enablers*	*Screening logsIssues registerSite meetings* ***Recruitment surveys***	Why did individuals agree to participate? Why not?What were the characteristics of individuals who agreed to participate? Did this suggest any bias?Did patients have trust in CGA, in providers and in the health service
***Delivery to individuals: what was delivered to the individual as the CGA intervention?*** *MRC domain: Implementation*	*Number and characteristics of participantsTiming, place, duration and documentation of CGAs* ***Redacted CGAsInterviews of health professionalsInterviews of patients and caregivers***	What form did the CGA take and what were the CGA components?To what extent was CGA multidimensional?To what extent was there adequate ‘dose’?To what extent was the assessment ‘structured’?To what degree was a multidisciplinary process employed?Did goal-setting form part of CGA?To what extent was collateral history and multisource feedback included?To what extent did the patients and caregivers agree with the management plan? To what extent was shared decision-making evident?Was goal setting linked to BCT?What was the fidelity with which the behaviour change techniques were implemented?Was there evidence of formulation of a management plan?How was communication structured?How did patients perceive the intervention?How did facilitation of CGA through nephrologists and research nurses change outcomes?What factors facilitate maintenance of CGA within and out of the trial setting?Was effective clinical leadership evident in care coordination?Was care coordination and follow-up present? To what extent was there regular review?
***Response of individuals and maintenance over time: how did individuals perceive the intervention and how did their behaviour change?*** *MRC domains: Acceptability, and barriers and enablers*	***Recruitment surveysInterviews of patients and caregivers***	Did individual behaviour change after CGA?How acceptable was the intervention?Did patients and caregivers have a view of the health issues and management plan that was congruent with that of the geriatrician?Did CGA foster self-management?Did individuals have trust in the service providers and health service?To what extent were families and caregivers involved?
***What are the unintended consequences of the intervention? What unforeseen outcomes of the intervention were observed, both wanted and unwanted?*** *MRC domains: Unintended consequences*	***Interviews of health professionalsInterviews of patients and caregivers***	What were the perceived outcomes/effects of CGA that were not expected or monitored in the main trial paper?What are the unintended consequences (good and bad) of providing CGA in the outpatient setting for frail older adults with CKD?What are the perceived barriers and harms of CGA in this setting, and how do these perceptions impact on outcomes?
***What theory can explain how CGA works? What are the causal mechanisms at play?*** *MRC domains: casual mechanism, barriers and enablers, and context*	***Redacted CGAsInterviews of health professionalsInterviews of patients and caregivers***	What components of CGA were perceived to be of benefit?What were the impediments to positive outcomes from CGA?What components (of the intervention, implementation or context) were associated with positive or negative outcomes?Do the assumptions made about causal mechanisms of how CGA impacts goal attainment add up, with reference to the logic model developed in the initial stage of the research?What are the important aspects which, if left out, undermine the efficacy of the CGA for older adults with CKD?
***What is the broader context in which the trial and CGA was delivered? How did context change how the trial was implemented and delivered, and how did this interact with the active components of the intervention to modify the outcome?*** *MRC domains: context, and barriers an enabler*	***Site meetings*** *Issues register* ***Redacted CGAs*** *Health diariesInterviews of health professionalsInterviews of patients and caregivers*	To what extent were clusters already providing CGA to these patients?Was there evidence of clinical leadership at an organisation level that influenced the implementation?Were the necessary resources available (time, money human, data systems, transport) to allow the success of CGA?Was there evidence of avenues and processes for communication and information sharing between MDT?Were patients involved in service design?How did the local organisational and health service structure, culture and local relationships impact on implementation and did they interact with causal mechanism to modify outcomes?What are the barriers and enablers to CGA effectiveness in the trial context and how reflective are these of those seen outside of the trial setting?How involved were GPs in the provision of CGA?How was billing/reimbursement structured at the site?

This uses the framework in Grant *et al*^25^ and addresses the process evaluation domains set out in the MRC guidance.^24^ For the data generation methods, those done as part of the main trial are highlighted in italics, whereas those that will be completed specifically for the process evaluation are in bold italics.

BCTbehaviour change techniqueCGAcomprehensive geriatric assessmentGPgeneral practitionerMDTmultidisciplinary teamMRCMedical Research Council

We aim to include all 16 sites where the GOAL trial is conducted in the process evaluation. These are in various locations around Australia, in New South Wales, Queensland, South Australia, Tasmania, Victoria and Western Australia. A mix of inner and outer metropolitan and regional sties will be included, although notably there are no rural locations. This allows consideration to be given to a range of different perspectives, and examination of how varying contextual factors, implementation processes and patient characteristic impact on trial outcomes.

### Development of programme theory

A narrative literature review will be conducted to generate hypotheses about the causal mechanisms at play in the efficacy of CGA and how implementation of CGA might be affected by context to augment outcomes. Through an iterative process of feedback and reflection between the authors, this will lead to the development of a hypothesised programme theory of how CGA might work in the outpatient setting.^24^

**Figure 2:**
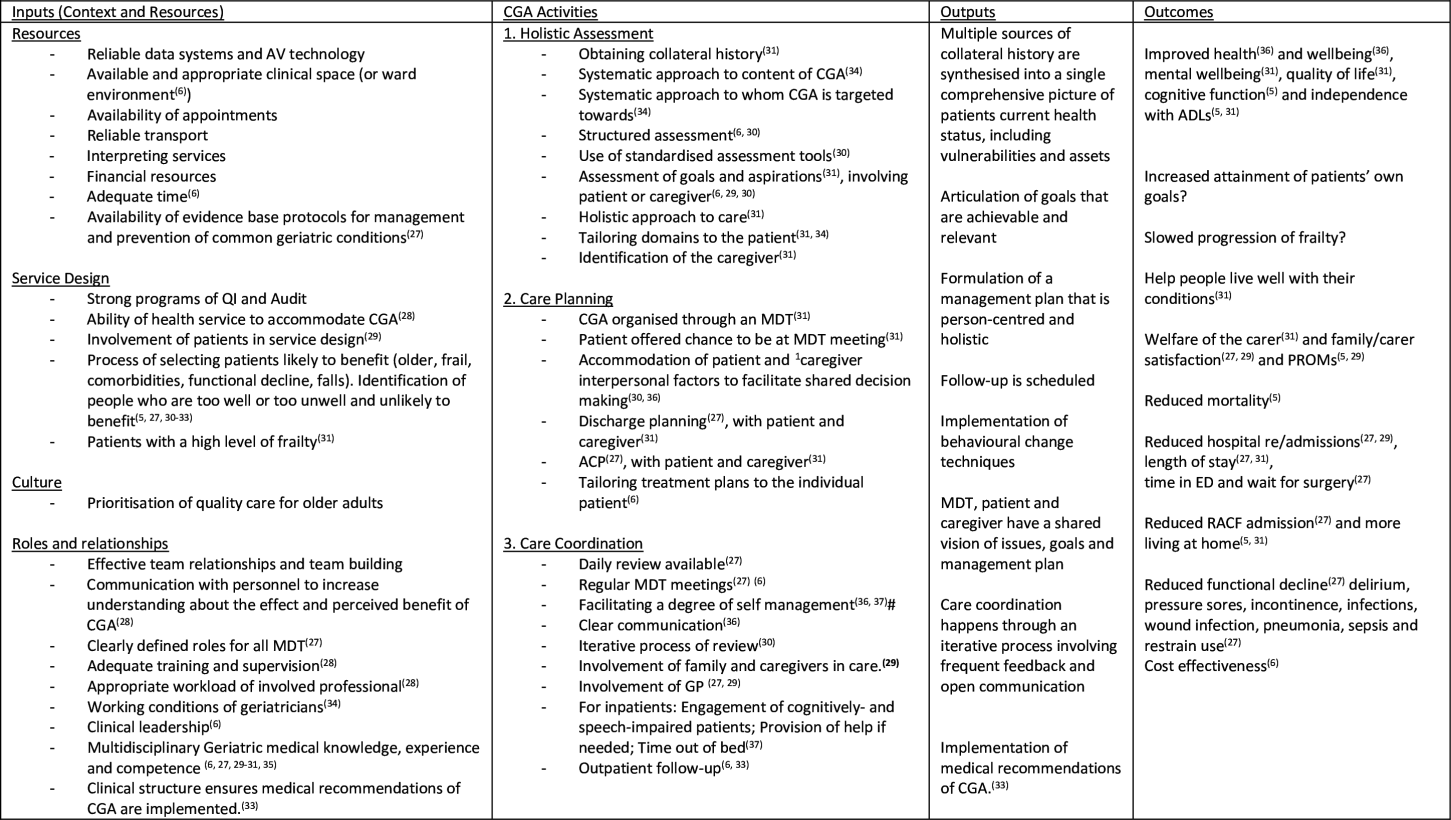
Logic model of CGA developed from literature review. ACP, advanced care planning; ADL, activities of daily living; AV, audio-visual; CGA, comprehensive geriatric assessment; ED, emergency department; GP, general practitioner; MDT, multidisciplinary team, PROM, patient-reported outcome measure; QI, quality improvement; RACF, residential-aged care facility.

In organising our programme theory in such a way that can guide collection of data, we will develop a logic model that accounts for the domains in the MRC Guidance to facilitate data analysis and coding.^23 24^ An early version of this logic model is presented in figure 2.

To date, an early programme theory has been developed, which will be further developed. We propose that CGA works through an iterative process of assessment that is holistic and takes account of the ‘whole person’. It includes development of a management plan and care coordination, both of which require a person-centred approach, interdisciplinary working and a focus on mutually agreed goals of care. CGA works when goals that are personally meaningful to the patient are framed so that a coordinated multidisciplinary management plan, that is, acceptable to the patient, can be organised. The multidisciplinary team, having clear roles and effective working relationships, work with the patient to achieve goals. This means the patient and caregiver perceive value in the intervention and trust the assessments of the multidisciplinary providers. Contextual inputs, such as access to support from the executive, adequate funding, physical resources and knowledge acquisition mean that treatment plans, can be executed with effectiveness. Communication, interprofessionally and between the patient and health professionals, is clear and effective meaning that all members are ‘on the same page’.

### Generation of research questions

The development of the programme theory allowed the generation of research questions that could be tested during the process evaluation. These research questions relate to CGA delivered during the trial as well as broader questions of how CGA might work in varying contexts. The process evaluation will be flexible enough to allow an iterative process of reflection and discussion, so that new learnings offered through the study can reinform and strengthen the underlying programme theory and allow generation of new research questions. The way in which these research questions link with MRC guidance, and how they have led to the specific questions in our evaluation work, are detailed in table 1.^24 25^

### Generation of data

Data will be generated from several sources

#### Implementation data will be collected after recruitment has closed at each site (ie, last patient, first visit)

1. Recruitment discussions: Minutes of site meetings and recruitment discussions, by trial staff will be analysed. These minutes will be recorded by the central coordinating centre during the trial and will not be transcribed by the process evaluation teams. A list of factors that facilitate or hinder recruitment will be documented. Difficulties that sites encounter recruiting individuals will also be discussed and recorded in regular meetings between sites and the clinical trial team. All data will be analysed and presented in a deidentified format.
2. Feasibility surveys were conducted by sites prior to being recruited to the trial. No identifying data will be shared or included in the analysis.
3. Recruitment survey: Principal investigators and research coordinators will be sent a survey about processes, facilitators and challenges with patient recruitment. This will be used in conjunction with interviews (below) to assess recruitment processes but will only be analysed and presented in a deidentified format. A copy of the recruitment surveys for principal investigators and research coordinators is available as onlinesupplemental appendices A  B.
4. Screening logs: These are kept by the research coordinators and include information such as a number of patients screened, approached and enrolled. They will be analysed in a deidentified format.
5. Trial issues register: This is kept by the central coordinating centre as a summary of all process issues identified during the trial. This will include issues related to recruitment, trial fidelity, communication issues, unintended consequences and barriers and enablers to the implementation of CGA at the site.

#### Redacted CGA letters

Intervention sites will provide written records of five CGA medical letters (per site), with redaction of identifying data. These will be convenience sampled. They will be analysed descriptively, using manual content analysis to extract the key domains assessed, multidisciplinary involvement and other key components such as goal-setting and follow-up. They will also be analysed qualitatively, to explore how geriatricians described the identified issues, patient goals and patient concerns, using a reflexive thematic analysis.^26 27^

#### Semistructured interviews with purposively sampled patients and their caregivers, and selected health professionals involved in the GOAL trial

1. Sampling and sample size: We will purposively sample health professionals, patients and caregivers for semistructured interviews. Interviewers of health professionals and patients will be conducted separately. All patients and caregivers who participate in the main trial will be eligible to interview. An effort will be made to capture a broad range of patients, with varying degrees of age (including those >85 years), frailty (including those with Frailty Index >0.4) and medical comorbidities.^28 29^ Of note, the GOAL trial protocol excludes patients with significant cognitive impairment who cannot consent for themselves. However, trial patients with mild cognitive impairment will be included. Given that we will rely on approached patients to consent to the interviews, convenience sampling will also be necessary, in addition to the purposive sampling described above. We will attempt to include a mix of geriatricians, nephrologists, research coordinators, trial investigators and administrative staff. If available, we will also interview multidisciplinary health professionals. We will aim for at least four patient and one caregiver interview per intervention site, two patient or caregiver interview from each control site and a total of 20 health professional interviews, continuing until thematic saturation is achieved.
2. Recruitment: Recruitment for the patient and caregiver interviews will commence when each site has completed all follow-up for all patients (ie, when the last patient at that site has completed their 12-month follow-up). Recruitment for health professionals will occur when all sites have completed all follow-up. This decision was made to prevent contamination and bias from the process evaluation affecting the main trial outcomes.
3. Consent for interviews: Written consent will be obtained for all participants interviewed. Consent for the interviews is not necessary for patient participation in the main trial.
4. Interview processes: Interviews will be conducted via audio-visual communication or telephone and will be audio-recorded using a digital recording device. Field notes may also be taken. Interview questions will be open-ended and phrased to encourage patients to discuss their own experiences and opinions. Medical and research jargon will be avoided. No repeat interviews will be carried out. Each interview will take approximately 30–60 min. Transcripts will not be returned to participants. No non-participants will be present for the interview. Interviews of caregivers can be conducted individually or dyadically.
5. Interview guides: Copies of the interview guides for research coordinators, geriatricians, nephrologists, patients and caregivers are available as online supplemental appendices C-G.

### Analysis of data

All implementation data (issues register, recruitment emails, trial site meetings) will be logged into spreadsheets. Descriptive statistics will be used to summarise issues noted in the process data (issues register as well as recruitment discussions) and descriptive data in the CGA letters. Quantitative data about the interview participants will also be described descriptively.

The interviews will be recorded and transcribed verbatim. Pseudonyms will be used in place of patient names and as such the stored information will be in a deidentified format. All identifiable data will be stored on password-protected files. The signed consent forms for patients and caregivers will be stored by the central coordinating centre while those for the health professionals will be stored by a member of the process evaluation team.

Qualitative data, from CGA letters, process data and semistructured interviews will be analysed using a reflexive thematic analysis, underpinned by an interpretivist epistemological position, using both deductive and inductive approaches.^27 30^ The deductive component will be informed by the programme theory and logic model developed in the first stage of the process evaluation. STF and one other team member will complete data coding. A copy of the coding tree will be provided with the published results. These will then be synthesised to further develop the programme theory that takes account of potential causal mechanisms. Participants will be invited to provide feedback on the findings. All qualitative data will be analysed with NVivo software and presented in a narrative format with excerpts of interviews included to illustrate key points.^31^

### Reporting guidelines

The Standards for Quality Improvement Reporting Excellence guidelines will be considered when reporting the results of the process evaluation.^32^ The qualitative components of this work, in particular the interviews, will be reported according to the Consolidated criteria for Reporting Qualitative research guidelines.^33^

### Patient and public involvement

Patient and public involvement is facilitated through the GOAL Trial Consumer Advisory Board.

## Ethics and dissemination

Findings from this process evaluation will be published in academic journals. There will be a clear linkage to the main trial paper. Results will also be presented at scientific conferences. Feedback to consumers will be facilitated through the central coordinating centre, in consultation with the GOAL trial consumer advisory board.

Ethics approval for process evaluation, along with the main trial, was granted by the Metro South Hospital and Health Service—Metro South Human Research Ethics Committee (HREC/2020/QMS/62883). The study has received local governance approvals at each of the participating sites. Protocol amendments are submitted to and approved by the ethics committee prior to implementation.

## supplementary material

10.1136/bmjopen-2023-076328 online supplemental file 1

10.1136/bmjopen-2023-076328 online supplemental file 2

10.1136/bmjopen-2023-076328 online supplemental file 3

10.1136/bmjopen-2023-076328 online supplemental file 4

10.1136/bmjopen-2023-076328 online supplemental file 5

10.1136/bmjopen-2023-076328 online supplemental file 6

10.1136/bmjopen-2023-076328 online supplemental file 7
