# Supplementary material for: Protocol for the process evaluation of the GOAL trial: investigating how comprehensive geriatric assessment (CGA) improves patient-centred goal attainment in older adults with chronic kidney disease in the outpatient setting
Source: BMJ Open. 2024 Aug 1;14(8):e076328. doi: 10.1136/bmjopen-2023-076328 (PMC11298742; doi:10.1136/bmjopen-2023-076328)
Supplement: online supplemental file 1 [file bmjopen-14-8-s001.pdf]

GOAL Trial Recruitment Survey - PI

Start of Block: Default Question Block

Q1 The GOAL-CKD Trial is a cluster randomised controlled trial investigating whether comprehensive geriatric assessment can allow frail older people with chronic kidney disease to better achieve their treatment goals.

This survey is about your experiences of patient recruitment, and is part of the process evaluation of the GOAL-CKD Trial.

The GOAL-CKD Trial, including the process evaluation component, has received ethics approval through
Metro South Hospital and Health Service - Metro South Human Research Ethics Committee (HREC/2020/QMS/62883).

Participation in this survey is voluntary. You will not be penalised if you don't complete this survey, and your involvement in this survey does not change or affect your involvement in the GOAL-CKD Trial more broadly.

It would be helpful for you to include your name and site ID when completing this survey. However, this is not necessary and it is ok if you prefer not to include these.

We anticipate this survey will take 5-10 minutes to complete.

If you have any questions about this survey please contact Dr Sarah Fox at sarah.fox@uq.edu.au or the GOAL Trial coordinators at goal@uq.edu.au

Thank you for your contribution to this survey and for your involvement in the GOAL-CKD Trial more broadly.

Q2 Was your Site involved in the GOAL-CKD trial?

- ○ Yes (1)
- ○ No (2)
- ○ Unsure (99)

Page Break

Display This Question:

If Was your Site involved in the GOAL-CKD trial? = Yes

Q3 What is your Site ID/Site Name?
Please note that this information is helpful but not necessary. Even if you do not want to provide your Site ID, we would be very grateful if you completed the other questions in the survey.

____

Display This Question:

If Was your Site involved in the GOAL-CKD trial? = Yes

Q4 Was your Site an *Intervention* or *Control* Site?

◯ Control (1)

◯ Intervention (2)

◯ Unsure (99)

Display This Question:

If Was your Site involved in the GOAL-CKD trial? = Yes

Q5 What was your role in the GOAL-CKD Trial?

◯ Principal Investigator (PI) - Geriatrician (1)

◯ Principal Investigator (PI) - Nephrologist (2)

◯ Other (please specify) (99) ____

Page Break

Display This Question:

If Was your Site involved in the GOAL-CKD trial? = Yes

X→

Q6 Did your Site (hospital or health service) already have an outpatient geriatrician clinic *prior* to GOAL?

○ Yes (1)

○ No (2)

○ Unsure (99)

Display This Question:

If Was your Site involved in the GOAL-CKD trial? = Yes

X→

Q7 Which clinicians were primarily looking after frail older *outpatients* with Chronic Kidney Disease prior to the GOAL-CKD trial?

○ Geriatricians (1)

○ Nephrologists (2)

○ Both geriatricians and nephrologists (3)

○ Other (Please specify) (4) ____

○ Unsure (99)

Display This Question:

If Was your Site involved in the GOAL-CKD trial? = Yes

X→

Q8 In your opinion, to what extent are the patients your site *recruited for the GOAL-CKD Trial* representative of *all frail patients* with CKD at your hospital/health service?

- ○ Very Representative (1)
- ○ Somewhat Representative (2)
- ○ Somewhat Unrepresentative (3)
- ○ Very Unrepresentative (4)
- ○ Unsure (99)

Display This Question:

If Was your Site involved in the GOAL-CKD trial? = Yes

And In your opinion, to what extent are the patients your site recruited for the GOAL-CKD Trial repre... != Very Representative

Q9 In what way were patients recruited for the GOAL Trial *NOT representative* of all frail older adults with CKD at your Site (hospital/health service)?

____

Page Break

Display This Question:

If Was your Site an Intervention or Control Site? = Intervention

And Was your Site involved in the GOAL-CKD trial? = Yes

Q10 How many geriatricians were involved in providing Comprehensive Geriatric Assessment as part of the GOAL-CKD Study at your Site?

- ○ 1 (1)
- ○ 2 (2)
- ○ 3 (3)
- ○ 4 (4)
- ○ >4 (5)
- ○ Unsure (99)

Display This Question:

If Was your Site involved in the GOAL-CKD trial? = Yes

And Was your Site an Intervention or Control Site? = Intervention

Q11 How easy was it to find geriatricians to provide CGA for the trial?

- ○ Extremely difficult (1)
- ○ Somewhat difficult (2)
- ○ Neither easy nor difficult (3)
- ○ Somewhat easy (4)
- ○ Extremely easy (5)
- ○ Unsure (99)

Display This Question:

If Was your Site an Intervention or Control Site? = Intervention

And Was your Site involved in the GOAL-CKD trial? = Yes

X→

Q12 How easy was it to find clinic space (room availability) for geriatricians to provide CGA as part of the GOAL Trial?

- ◯ Extremely difficult (1)
- ◯ Somewhat difficult (2)
- ◯ Neither easy nor difficult (3)
- ◯ Somewhat easy (4)
- ◯ Extremely easy (5)
- ◯ Unsure (99)

Page Break

Display This Question:

If Was your Site involved in the GOAL-CKD trial? = Yes

X→

Q13 To what degree did the Covid-19 pandemic negatively impact on recruitment for the GOAL-CKD Trial?

- ○ Not at all (1)
- ○ A small amount (2)
- ○ A moderate amount (3)
- ○ A great amount (4)
- ○ A very great amount (5)
- ○ Unsure (99)

Display This Question:

If Was your Site involved in the GOAL-CKD trial? = Yes

Q14 How did the Covid-19 pandemic negatively impact recruitment for the trial?

____

Display This Question:

If Was your Site involved in the GOAL-CKD trial? = Yes

And Was your Site an Intervention or Control Site? = Intervention

Q15 How did you manage outpatient scheduling to ensure clinic availability for geriatricians to provide CGA as part of the GOAL Trial?

____

Page Break

Display This Question:

If Was your Site involved in the GOAL-CKD trial? = Yes

Q16 How supportive were *nephrologists* at your site of patients being involved in the GOAL Trial?

- ○ Very Supportive (1)
- ○ Somewhat Supportive (2)
- ○ Somewhat Unsupportive (3)
- ○ Very Unsupportive (4)
- ○ Unsure (99)

Display This Question:

If Was your Site involved in the GOAL-CKD trial? = Yes

Q17 How supportive were *geriatricians* at your site of patients being involved in the GOAL Trial?

- ○ Very Supportive (1)
- ○ Somewhat Supportive (2)
- ○ Somewhat Unsupportive (3)
- ○ Very Unsupportive (4)
- ○ Unsure (99)

Display This Question:

If Was your Site involved in the GOAL-CKD trial? = Yes

Q18 How supportive were *hospital executive/management* at your site of patients being involved in the GOAL Trial?

- ○ Very Supportive (1)
- ○ Somewhat Supportive (2)
- ○ Somewhat Unsupportive (3)
- ○ Very Unsupportive (4)
- ○ Unsure (99)

Page Break

Display This Question:

If Was your Site involved in the GOAL-CKD trial? = Yes

Q19 What factors *supported or assisted* patient recruitment at your Site?

____

Display This Question:

If Was your Site involved in the GOAL-CKD trial? = Yes

Q20 From your perspective, what were the *barriers or challenges* to patient recruitment at your Site?

____

Display This Question:

If Was your Site involved in the GOAL-CKD trial? = Yes

Q21 In retrospect, what could have been *done differently* (trial design, trial management, site organisation etc) to improve recruitment?

____

Display This Question:

If Was your Site involved in the GOAL-CKD trial? = Yes

Q22 Is there anything else you would like to say about recruitment for the GOAL Trial?

____

Display This Question:

If Was your Site involved in the GOAL-CKD trial? = Yes

Q23 If you are happy to leave your name, please enter it here:

____

End of Block: Default Question Block
