# Supplementary material for: Protocol for the process evaluation of the GOAL trial: investigating how comprehensive geriatric assessment (CGA) improves patient-centred goal attainment in older adults with chronic kidney disease in the outpatient setting
Source: BMJ Open. 2024 Aug 1;14(8):e076328. doi: 10.1136/bmjopen-2023-076328 (PMC11298742; doi:10.1136/bmjopen-2023-076328)
Supplement: online supplemental file 2 [file bmjopen-14-8-s002.pdf]

GOAL Trial Recruitment Survey - Research Coordinators

Start of Block: Default Question Block

Q1 The GOAL-CKD Trial is a cluster randomised controlled trial investigating whether comprehensive geriatric assessment (CGA) can allow frail older people with chronic kidney disease to better achieve their treatment goals.

This survey is about your experiences of patient recruitment, and is part of the process evaluation of the GOAL-CKD Trial.

The GOAL-CKD Trial, including the process evaluation component, has received ethics approval through
Metro South Hospital and Health Service - Metro South Human Research Ethics Committee (HREC/2020/QMS/62883).

Participation in this survey is voluntary. You will not be penalised if you don't complete this survey, and your involvement in this survey does not change or affect your involvement in the GOAL-CKD Trial more broadly.

It would be helpful for you to include your name and Site ID when completing this survey. However, it is not necessary and it is ok if you prefer not to include these.

We anticipate this survey will take 5-10 minutes to complete.

If you have any questions about this survey please contact Dr Sarah Fox at sarah.fox@uq.edu.au or the GOAL Trial coordinators at goal@uq.edu.au

Thank you for your contribution to this survey and for your involvement in the GOAL-CKD Trial more broadly.

Q2 Was your Site involved in the GOAL-CKD trial?

- ○ Yes (1)
- ○ No (2)
- ○ Unsure (99)

Page Break

Display This Question:

If Was your Site involved in the GOAL-CKD trial? = Yes

Q3 What is your Site ID/Site Name? (optional)
Please note that this information is helpful but not necessary. Even if you do not want to provide your Site ID, we would be very grateful if you completed the other questions in the survey.

____

Display This Question:

If Was your Site involved in the GOAL-CKD trial? = Yes

X→

Q4 Was your Site an *Intervention* or *Control* Site?

○ Control (1)

○ Intervention (2)

○ Unsure (99)

Display This Question:

If Was your Site involved in the GOAL-CKD trial? = Yes

X→

Q5 What was your role in the GOAL-CKD Trial?

○ Research Coordinator or Research Nurse (1)

○ Other (please specify) (99) ____

Page Break

Display This Question:

If Was your Site involved in the GOAL-CKD trial? = Yes

And Was your Site an Intervention or Control Site? = Intervention

Q6 How many geriatricians were involved in providing Comprehensive Geriatric Assessment as part of the GOAL-CKD Study at your Site?

- ◯ 1 (1)
- ◯ 2 (2)
- ◯ 3 (3)
- ◯ 4 (4)
- ◯ >4 (5)
- ◯ Unsure (99)

Display This Question:

If Was your Site involved in the GOAL-CKD trial? = Yes

And Was your Site an Intervention or Control Site? = Intervention

Q7 How easy was it to find geriatricians to provide Comprehensive Geriatric Assessment (CGA) at your site?

- ◯ Very Easy (1)
- ◯ Somewhat Easy (2)
- ◯ Somewhat Difficult (3)
- ◯ Very Difficult (4)
- ◯ Unsure (99)

Display This Question:

If Was your Site involved in the GOAL-CKD trial? = Yes

Q8 From *where* were trial participants predominantly recruited?

- ☐ Dialysis outpatients (1)
- ☐ Renal outpatient department (Non-Dialysis) (2)
- ☐ Inpatients - Renal ward (3)
- ☐ Inpatients - Other wards (4)
- ☐ Emergency Department (5)
- ☐ General Practice (GP) (6)
- ☐ Other (Please specify) (99) ____

Display This Question:

If Was your Site an Intervention or Control Site? = Intervention

And Was your Site involved in the GOAL-CKD trial? = Yes

Q9 Did patients require a GP referral to have a Geriatrician assessment (CGA) as part of this trial?

- ○ Yes (1)
- ○ No (2)
- ○ Unsure (99)

Page Break

Display This Question:

If Was your Site involved in the GOAL-CKD trial? = Yes

Q10 To what degree did the Covid-19 pandemic negatively impact on recruitment for the GOAL-CKD Trial?

- ○ Not at all (1)
- ○ A small amount (2)
- ○ A moderate amount (3)
- ○ A great amount (4)
- ○ A very great amount (5)
- ○ Unsure (99)

Display This Question:

If Was your Site involved in the GOAL-CKD trial? = Yes

And To what degree did the Covid-19 pandemic negatively impact on recruitment for the GOAL-CKD Trial? != Not at all

Q11 In what way did the Covid-19 pandemic negatively impact recruitment for the trial?

____

Page Break

Display This Question:

If Was your Site involved in the GOAL-CKD trial? = Yes

Q12 How much did transport requirements (e.g. transport to trial appointments) negatively impact recruitment at your site?

- None at all (1)
- A little (2)
- A moderate amount (3)
- A lot (4)
- A great deal (5)
- Unsure (99)

Display This Question:

If Was your Site involved in the GOAL-CKD trial? = Yes

Q13 To what extent were time constraints for the *patient* (e.g. time for appointments) a reason for patients not wanting to participate in the trial?

- None at all (1)
- A little (2)
- A moderate amount (3)
- A lot (4)
- A great deal (5)

Display This Question:

If Was your Site involved in the GOAL-CKD trial? = Yes

Q14 To what extent were time constraints for the *caregiver* (e.g. time for appointments) a reason for patients not wanting to participate in the trial?

- None at all (1)
- A little (2)
- A moderate amount (3)
- A lot (4)
- A great deal (5)
- Unsure (99)

Display This Question:

If Was your Site involved in the GOAL-CKD trial? = Yes

Q15 To what extent were costs associated with parking a reason for patients not wanting to participate in the trial?

- None at all (1)
- A little (2)
- A moderate amount (3)
- A lot (4)
- A great deal (5)
- Unsure (99)

Page Break

Display This Question:

If Was your Site involved in the GOAL-CKD trial? = Yes

Q16 What factors *supported or assisted* patient recruitment at your Site?

____

Display This Question:

If Was your Site involved in the GOAL-CKD trial? = Yes

Q17 From your perspective, what were the *barriers or challenges* to patient recruitment at your Site?

____

Display This Question:

If Was your Site involved in the GOAL-CKD trial? = Yes

Q18 In retrospect, what could have been *done differently* (trial design, trial management, site organisation etc) to improve recruitment?

____

Display This Question:

If Was your Site involved in the GOAL-CKD trial? = Yes

Q19 Is there anything else you would like to say about recruitment for the GOAL Trial?

____

Display This Question:

If Was your Site involved in the GOAL-CKD trial? = Yes

Q20 If you are happy to leave your *name*, please enter it here:

____

End of Block: Default Question Block
