# Supplementary material for: Protocol for the process evaluation of the GOAL trial: investigating how comprehensive geriatric assessment (CGA) improves patient-centred goal attainment in older adults with chronic kidney disease in the outpatient setting
Source: BMJ Open. 2024 Aug 1;14(8):e076328. doi: 10.1136/bmjopen-2023-076328 (PMC11298742; doi:10.1136/bmjopen-2023-076328)
Supplement: online supplemental file 3 [file bmjopen-14-8-s003.pdf]

Interview Guide for Research coordinators/Research staff/Administrative staff

Introduction

The interview is about your experience of the GOAL study, in which patients with chronic kidney disease saw a geriatrician in an outpatient clinic.

Thank you for discussing your experiences. You have a unique perspective in helping us understand what in the study worked well and what didn't. There are no right or wrong answers; your personal views and experiences are what interest me.

The decision to be involved in this interview is entirely up to you. If at any point there are questions you do not want to answer please let me know – you do not have to answer any question if you don't want to.

With your permission I would like to record our conversation today so that I can listen carefully and later on re-listen to the recording to extract the most useful aspects. We take your confidentiality very seriously. When we transcribe the interviews we will remove any details that might identify you. We then collate the responses from your interview and the interviews with other people. Your name will never be published as one of the individuals who participated in the interview part of this research and it will not be possible to identify you from any material published from this interview.

Is it ok with you if I record our conversation today?

<Start recording>

1. **Health professional background**
   Firstly, could you please briefly describe your previous experience in working with older people?
   Prompts:
   - Did you already have experience of working with the renal physicians and geriatricians in this hospital?
   - Did you have experience in the outpatient clinic here?
   - What role are were you in prior to, and at the time of, the GOAL trial

2. **Role in the GOAL Study**
   Do you remember the GOAL Study? Are you able to talk with me about where you fit into the GOAL Study and what your role in it was?
   Prompts:
   - role in recruitment
   - role in data collection
   - role in getting geriatricians/stakeholders on board

3. **Perceived value of the GOAL trial**
   Why did you agree to participate in the GOAL trial?
   Prompts:
   - What were the foreseen advantages of you or your site being involved?
   - Were there things that you were worried would be difficult when you were deciding whether to participate?

4. **Implementation of CGA, including barriers and enablers**
   Can you describe how the CGA, the assessment where the patient was seen by the geriatrician, was incorporated into the outpatient clinic?
   Prompts:
   - How did it run logistically?
   - Were their any challenges in setting it up?
   - Was it difficult to get referrals from GPs/specialists?
   - How it was billed
   - Was it hard to find clinic space?
   - Was it hard to find a geriatrician?

5. **Recruitment**
   How were patients recruited and how well did this process of recruitment run?
   Prompts:
   - Do you think the patients who were included were representative of most frail older people with CKD?
   - Were patients willing? Why did some people said no?
   - How onerous was recruitment? Was it hard to get nephrologists on board? Was it hard to coordinate the geriatric and nephrology care?

6. **GAS**
   What was your experience of doing GAS with the patient?
   Prompts:
   - What were the good and bad things about this?
   - How much did the GAS you did in practise reflect what you learnt about GAS at the start of the study?
   - What were the challenges and what do you think could be improved?

7. **Barriers and Enablers and Maintenance**
   What were the barrier and enablers to embedding CGA into the care of older adults with CKD? Did this change over time?
   Prompts:
   - Did you change your processes over the 2 years that the trial was running? If so why?
   - What were the barriers and challenges to recruitment, implementation and patient care that you face
   -

8. **CGA Acceptability and Value**
   Based on your experiences with this study, do you think it would be good to have a geriatrician integrated into the care team for older frail patients with chronic kidney disease? If yes, why; if no, why not? Prompts:
   - Positive things: benefits of in-depth assessment of goals of patients; good to share the care for frail patients
   - Negative things: decision making can be done with another health care professional already; patients feel too unwell or overwhelmed at the time; kidney team is taking good care of the patients; patients already have multiple providers - adding the geriatrician makes it more complex; geriatrician comes

and goes but we must then action the prescriptions/plans, that is the hard part; geriatricians explanations come too late; patients already set their mind on a path forward etc.)

- Did you speak to others about the intervention, and would you recommend other teams to integrate a comprehensive geriatric assessment into their care pathways?
-

9. **Wellbeing**
   Did the intervention/study impact your own work and wellbeing? If yes, in what way? If no, why not?

10. **Other**
    Would you have any other comments to share of the overall experience of the intervention over time in your clinic and how the patients, the team or you were impacted by it?

Thank you very much for your time.
