# Supplementary material for: Protocol for the process evaluation of the GOAL trial: investigating how comprehensive geriatric assessment (CGA) improves patient-centred goal attainment in older adults with chronic kidney disease in the outpatient setting
Source: BMJ Open. 2024 Aug 1;14(8):e076328. doi: 10.1136/bmjopen-2023-076328 (PMC11298742; doi:10.1136/bmjopen-2023-076328)
Supplement: online supplemental file 4 [file bmjopen-14-8-s004.pdf]

Interview Guide for Geriatricians

Introduction

The interview is about your experience of the GOAL study, in which patients with chronic kidney disease saw a geriatrician in an outpatient clinic.

Thank you for discussing your experiences. You have a unique perspective in helping us understand what in the study worked well and what didn't. There are no right or wrong answers; your personal views and experiences are what interest me.

The decision to be involved in this interview is entirely up to you. If at any point there are questions you do not want to answer please let me know – you do not have to answer any question if you don't want to.

With your permission I would like to record our conversation today so that I can listen carefully and later on re-listen to the recording to extract the most useful aspects. We take your confidentiality very seriously. When we transcribe the interviews we will remove any details that might identify you. We then collate the responses from your interview and the interviews with other people. Your name will never be published as one of the individuals who participated in the interview part of this research and it will not be possible to identify you from any material published from this interview.

Is it ok with you if I record our conversation today?

<Start Recording>

1. **Health professional background**
   Firstly, could you please briefly describe your previous experience in working with frail older people with chronic kidney disease? And also your hospitals processes and programs for frail older adults with CKD, that were already up and running prior to this study starting?
   Prompts:
   - Did you already have experience of working with the renal physicians in this hospital?
   - Were you already seeing a lot of patients with CKD?
   - What were the relationships like between nephrology and geriatric medicine?
   - Did you have geriatric clinics here in the OP prior to the study starting? Did you see many referrals from nephrologists in these clinics?
   - Who was looking after older adults with CKD? Geriatricians or nephrologists?

2. **Role in the GOAL Study**
   Do you remember the GOAL Study? Are you able to talk with me about where you fit into the GOAL Study and what your role in it was?
   Prompts:
   - role in leading/coordinating study
   - role in seeing patients
   - role in following up patients?
   - Assessment of eligibility/recruitment process?

- role in booking times for geriatricians/clinic rooms
- role in making sure the CGA and the GAS happened close together
- Were you a PI? Member of TSC?

3. Perceived value of the GOAL trial

Why did you agree to participate in the GOAL trial? Did you think that the intervention would be beneficial?

Prompts:

- What were the foreseen advantages of you or your site being involved?
- Were there things that you were worried would be difficult when you were deciding whether to participate?
- Did you think the trial would benefit patients?
- Did you think the trial/intervention would be beneficial for your site?
- What were you worried wouldn't work/might be difficult with the study?

4. Implementation

Can you describe how the CGA, was incorporated into the outpatient clinic? Prompts:

- How did it run logistically?
- Were their any challenges in setting it up?
- Was it difficult to get referrals from GPs/specialists?
- How it was billed
- Was it hard to find clinic space?
- How much did the CGA you provided in this trial reflect your real-world practice?
-

5. CGA Acceptability and Value

Based on your experiences with this study, do you think it would be good to have a geriatrician integrated into the care team for older frail patients with chronic kidney disease? If yes, why; if no, why not?

Prompts:

- Can you describe to me what it is about the CGA intervention, including what happened during the consultation and what the follow-up /review, that makes it beneficial or not??

-

6. Processes – Communication and Information Sharing

Can you describe the processes of communication and information sharing when providing CGA as part of the trial?

Prompts:

- How much did you communicate with the nephrologists?
- How did you communicate with the GP?
- How did you communicate with the MDT?
- Was the communication ongoing or just a single moment?
- How adequate was the medical record/information transfer system?

7. **Process – Goal setting and Care planning**
   Patients in this trial set goals (GAS).Did you discuss patients goals, set during the trial with them? If so is this different to your usual care? Did you discuss other goals?
   Prompts:
   - Did you have access to the frailty index and did this change the assessment?
   - Did you have access to the GAS and did this change the assessment?

8. **Processes – follow-up and review**
   What follow-up and review did you have for the patients you saw in the trial?
   Prompts:
   - Did you see patients again and follow-up their progress?
   - To what degree did you feel you took ownership of their care? And to what degree did most ownership stay with the nephrologist or GP?

9. **Barriers and Enablers**
   What things improved or hindered the delivery of CGA for patients with CKD?
   - What aspects of the hospital system/people with whom you work could have been improved?
   - Were there supports/people in place that made it easier to care for patients in this setting?
   - Were there supports/people in place that made it harder to care for people
   - What could be changed about the system in which you work to make CGA more accessible and more effective?
   - Were there any additional costs to your unit or to the patients or carers by integrating the intervention in routine care?

10. **Wellbeing**
    Did the intervention/study impact your own work and wellbeing? If yes, in what way? If no, why not?

11. **Other**
    Would you have any other comments to share of the overall experience of the intervention over time in your clinic and how the patients, the team or you were impacted by it?

Thank you very much for your time.
