# Supplementary material for: Protocol for the process evaluation of the GOAL trial: investigating how comprehensive geriatric assessment (CGA) improves patient-centred goal attainment in older adults with chronic kidney disease in the outpatient setting
Source: BMJ Open. 2024 Aug 1;14(8):e076328. doi: 10.1136/bmjopen-2023-076328 (PMC11298742; doi:10.1136/bmjopen-2023-076328)
Supplement: online supplemental file 5 [file bmjopen-14-8-s005.pdf]

Interview Guide for Nephrologists

Introduction

The interview is about your experience of the GOAL study, in which patients with chronic kidney disease saw a geriatrician in an outpatient clinic.

Thank you for discussing your experiences. You have a unique perspective in helping us understand what in the study worked well and what didn't. There are no right or wrong answers; your personal views and experiences are what interest me.

The decision to be involved in this interview is entirely up to you. If at any point there are questions you do not want to answer please let me know – you do not have to answer any question if you don't want to.

With your permission I would like to record our conversation today so that I can listen carefully and later on re-listen to the recording to extract the most useful aspects. We take your confidentiality very seriously. When we transcribe the interviews we will remove any details that might identify you. We then collate the responses from your interview and the interviews with other people. Your name will never be published as one of the individuals who participated in the interview part of this research and it will not be possible to identify you from any material published from this interview.

Is it ok with you if I record our conversation today?

<Start recording>

1. **Health professional background**
   Firstly, could you please briefly describe your previous experience in working with frail older people with chronic kidney disease? And also your hospitals processes and programs for frail older adults with CKD, that were already up and running prior to this study starting?
   Prompts:
   - What role are were you in prior to, and at the time of, the GOAL trial?
   - Were you already seeing a lot of patients with CKD who were older and frail?
   - What were the relationships like between nephrology and geriatric medicine?
   - Who was looking after older adults with CKD? Geriatricians or nephrologists?
   - Has it been difficult to have patients seen by a geriatrician in the past?
   - How established and effective were the care pathways for frail older adults with CKD, prior to this study commencing?

2. **Role in the GOAL Study**
   Do you remember the GOAL Study? Are you able to talk with me about where you fit into the GOAL Study and what your role in it was?
   Prompts:
   - Role in recruitment
   - Role in data collection
   - Role in getting geriatricians/stakeholders on board
   - Were you a PI? Member of TSC?
   -

3. **Perceived value of the GOAL trial**

Why did you agree to participate in the GOAL trial? Did you think that the intervention would be beneficial?

Prompts:

- What were the foreseen advantages of you or your site being involved?
- Were there things that you were worried would be difficult when you were deciding whether to participate?
- Did you think the trial would benefit patients?
- Did you think the trial/intervention would be beneficial for your site?
- What were you worried wouldn't work/might be difficult with the study?

4. **Implementation and Processes of CGA**

Can you describe how the CGA, the assessment where the patient was seen by the geriatrician, was incorporated into the outpatient clinic?

Prompts:

- Were their any challenges in setting it up?
- How much did you communicate with the geriatricians providing the CGA? Did you receive a letter from the geriatrician?Did you discuss the patient verbally either in person or over the phone
- How adequate was the information sharing between you and the geriatrician
- Did you continue to see patients who had been seen by geriatricians? Why or why not?
- Your interactions with patients and carers who received this intervention differed from the usual care pathway
- Did you change your management based on the geriatricians' recommendations?

5. **Recruitment**

How were patients recruited and how well did this process of recruitment run?

Prompts:

- Do you think the patients who were included were representative of most frail older people with CKD?
- Were patients willing? Why did some people said no?
- How did you decide who to screen/include in the trial? Did other trials running simultaneoulsy mean that the patients include in the GOAL trial were not representative?
-

6. **GAS**

Patients in this trial set goals(GAS).Did you discuss patients goals, set during the trial with them? If so is this different to your usual care?

7. **CGA Acceptability and Value**

Based on your experiences with this study, do you think it would be good to have a geriatrician integrated into the care team for older frail patients with chronic kidney disease? If yes, why; if no, why not? Prompts:

- Positive things: benefits of in-depth assessment of goals of patients; good to share the care for frail patients
- Negative things: decision making can be done with another health care professional already; patients feel too unwell or overwhelmed at the time;

kidney team is taking good care of the patients; patients already have multiple providers - adding the geriatrician makes it more complex; geriatrician comes and goes but we must then action the prescriptions/plans, that is the hard part; geriatricians explanations come too late; patients already set their mind on a path forward etc.)

- Did you speak to others about the intervention, and would you recommend other teams to integrate a comprehensive geriatric assessment into their care pathways?

8. **Barriers and Challenges**
   What were the challenges associated with patients receiving CGA as part of this trial?
   - What are the barriers in referring patients to geriatricians
   - Did the trial and geriatrician assessment increase or decrease your workload?

9. **Wellbeing**
   Did the intervention/study impact your own work and wellbeing? If yes, in what way? If no, why not?

10. **Other**
    Would you have any other comments to share of the overall experience of the intervention over time in your clinic and how the patients, the team or you were impacted by it?

Thank you very much for your time.
