# Supplementary material for: Protocol for the process evaluation of the GOAL trial: investigating how comprehensive geriatric assessment (CGA) improves patient-centred goal attainment in older adults with chronic kidney disease in the outpatient setting
Source: BMJ Open. 2024 Aug 1;14(8):e076328. doi: 10.1136/bmjopen-2023-076328 (PMC11298742; doi:10.1136/bmjopen-2023-076328)
Supplement: online supplemental file 6 [file bmjopen-14-8-s006.pdf]

Interview Guide for Patients

Introduction

The interview is about your experience of being a participant in the GOAL study. In this study you saw a geriatrician in the outpatient clinic.

Thank you for discussing your experiences. You have a unique perspective in helping us understand what in the study worked well and what didn't. There are no right or wrong answers; your personal views and experiences are what interest me.

The decision to be involved in this study is entirely up to you. If at any point there are questions you do not want to answer please let me know – you do not have to answer any question if you don't want to.

With your permission I would like to record our conversation today so that I can listen carefully and later on re-listen to the recording to extract the most useful aspects. We take your confidentiality very seriously. When we transcribe the interviews we will remove any details that might identify you. We then collate the responses from your interview and the interviews with other people. Your name will never be published as one of the individuals who participated in the interview part of this research and it will not be possible to identify you from any material published from this interview.

Is it ok with you if I record our conversation today?

<Start recording>

1. **Recruitment**
   Are you able to describe how you came to be involved in the GOAL study and why you agreed to be involved?
   Prompts
   - Why did you agree to participate in this study?
   - What were you hoping might be the positive outcomes?
   - Were there any factors that made your hesitant or unsure when deciding whether to participate?
   - How did the nephrologist or nurse frame the benefits/harms when talking about the study?

2. **Experience of CGA**
   As part of the trial, you had consultation with a geriatrician. Can you remember that assessment and consultation by the geriatrician and can you tell me how that went?
   Prompts
   - What did you understand would happen in the assessment?
   - Is what actually happened in the geriatrician appointment different to what you expected?
   - Did you discuss the goals you had previously identified?
   - What was the geriatrician most interested in?
   - What were the outcomes from that assessment?
   - Which parts of the assessment held the most value for you?

3. **Goal Attainment Scaling**

Do you remember doing the goal-setting with the research coordinator prior to seeing the geriatricians? How helpful was this and how did it frame what you discussed when you did see the geriatrician?

Prompts:

- Do you think there was value in doing this GAS? What was it?
- Were the goals meaningful and relevant to you?
- Did you discuss the goals with the geriatrician?
- Did you have a plan for how to achieve your goals, and did the consultation with the geriatrician assist with this?
- Did you discuss your health goals with the geriatrician? What was your experience of this? How did this impact on your treatment and wellbeing choices at the time, and over time?

4. **Role of MDT**

Did the appointment with the geriatrician lead to other assessments or appointments? E.g. , occupational therapist, social worker, counsellor or other doctor?

Prompts:

- Were these consultations useful?
- Were they important to reach your goal?
- Did you get a sense of the MDT being 'on the same page'
- Did you sense that the MDT were communicating with each other?

5. **Acceptability and value of CGA**

Would you like to have similar consultation with this geriatrician again? If yes, why; if no, why not?

Prompts:

- Did you feel that the geriatrician consultation added value? Was the consultation helpful?
- What were the positive aspects of the consultation?
- Was there anything that did not go so well for you in that conversation or that you would have liked to be done differently?
- What were the negative aspects of the consultation? E.g. care and decision making already happening with another team, too sick, too overwhelmed with appointments, seeing geriatrician too late, lack of continuity etc
- Were there any unexpected positive or negative outcomes of the assessment?
- Overall would you recommend this consultation to others?
- Did the consultant impact your quality of life?

6. **Barriers and Challenges**

Was there anything that made it difficult for you to participate in the geriatrician consultation?

- Was transport to the appointment difficult?
- Did the appointments take up too much time?
- Were there other reasons for why the conversation was not so helpful – maybe felt too unwell or overwhelmed at the time?
- Were there any additional costs to you because of the geriatrician assessment?

7. **Processes**

   What was it about the consultation that made it a positive or negative experience?

   Prompts:

   - Did the geriatrician involve you in the plan and decision-making?
   - Did the geriatrician understand your concerns?
   - Was communication adequate?
   - Was there good rapport with the geriatrician?
   -

8. **Wellbeing**

   Did the intervention/study impact your own work and wellbeing? If yes, in what way? If no, why not?

9. **Other**

   Would you like to make any other comments about your experience of being involved in the trial, or having the geriatrician assessment?

Any other comments before we close?

Thank you very much for your time. We really appreciate the effort you have gone to share your experiences and improve our knowledge about this trial.
