# Supplementary material for: Protocol for the process evaluation of the GOAL trial: investigating how comprehensive geriatric assessment (CGA) improves patient-centred goal attainment in older adults with chronic kidney disease in the outpatient setting
Source: BMJ Open. 2024 Aug 1;14(8):e076328. doi: 10.1136/bmjopen-2023-076328 (PMC11298742; doi:10.1136/bmjopen-2023-076328)
Supplement: Supplementary file 8 [file bmjopen-2023-076328.reviewer_comments.pdf]

PEER REVIEW HISTORY

BMJ Open publishes all reviews undertaken for accepted manuscripts. Reviewers are asked to complete a checklist review form (**http://bmjopen.bmj.com/site/about/resources/checklist.pdf**) and are provided with free text boxes to elaborate on their assessment. These free text comments are reproduced below.

ARTICLE DETAILS

**TITLE (PROVISIONAL)**	A Protocol for the Process Evaluation of the GOAL Trial: investigating how comprehensive geriatric assessment (CGA) improves patient-centred goal attainment in older adults with chronic kidney disease in the outpatient setting
**AUTHORS**	Fox, Sarah; Hubbard, Ruth; Valks, Andrea; Matsuyama, Misa; Kalaw, Emarene; Viecelli, Andrea; Aquino, Eunise; Johnson, David; Janda, Monika

VERSION 1 – REVIEW

**REVIEWER**	Asad Merchant<br>University of Toronto
**REVIEW RETURNED**	21-Jun-2023

**GENERAL COMMENTS**	The protocol paper might benefit from a more detailed description of the qualitative research methodological theory that is to be used to analyse the data, including an explanation of program theory.<br><br>Recruitment of patients is not clear - there do not appear to be specific inclusion criteria. Frailty index and age and co-morbidities are listed as characteristics rather than clear inclusion criteria.

**REVIEWER**	Carlijn Voorend<br>Leiden University Medical Center
**REVIEW RETURNED**	06-Jul-2023

**GENERAL COMMENTS**	Fox and collegues described the protocol of a study that aims to understand how CGA might be effective for older frail adults with CKD in the outpatient setting. They planned to conduct a process evaluation of the cluster randomised controlled GOAL trial (protocol published by Logan et al., 2023 in Trials). They specifically aim to investigate implementation, mechanisms of impact and context of the CGA intervention, which are the three key components of the MRC guidance for process evaluation of complex interventions. Amongst other resources (implementation and recruitment data, medical letters), the researchers aim to survey PIs and research coordinators, and conduct additional interviews with research coordinators, research staff, and administrative staff.<br><br>Fox and collegues described the protocol of a study that aims to understand how CGA might be effective for older frail adults with CKD in the outpatient setting. They planned to conduct a process evaluation of the cluster randomised controlled GOAL trial (protocol published by Logan et al., 2023 in Trials). They specifically aim to investigate implementation, mechanisms of impact and context of the CGA intervention, which are the three key components of the

	MRC guidance for process evaluation of complex interventions. Amongst other resources (implementation and recruitment data, medical letters), the researchers aim to survey PIs and research coordinators, and conduct additional interviews with research coordinators, research staff, and administrative staff.<br>First, I applaud the authors' plans to conduct an extensive structured process evaluation for the complex CGA intervention in CKD care. This study will contribute valuable information for integrating geriatric and nephrological care. I have some questions and remarks to consider for publishing the protocol.<br>1. The title is precise and informative, though long. The authors may want to consider omitting 'a pragmatic cluster randomised controlled trial' since this concerns the study design of the GOAL trial rather than the current process evaluation. They could also consider changing 'whether' for 'how', fitting the aim of the current evaluation study.<br><br>2. Abstract<br>a. The investigators may want to consider including a sentence on how outcomes of this process evaluation are assumed to benefit care/research.<br>b. Is it common for BMJ Open to include references in the abstract?<br>c. Have you considered if the STARI checklist or SQUIRE guideline would fit the purpose of reporting the outcomes of the process evaluation?<br><br>3. Introduction<br>a. (page 4, lines 25-35): Could you provide some sentences to inform the reader about the intervention of the GOAL Trial? (e.g. what CGA does it exist of, by whom is it conducted, where and how?)<br>b. In the GOAL-trial protocol, Logan et al describe "a process evaluation will be conducted [..] whether the intervention was delivered as intended, any issue or local barriers to invention delivery, and perceptions of the intervention by participants". In the introduction (final sentence) of the current manuscript the authors state: "we will be conducting a dedicated process evaluation of the GOAL trial, focusing on recruitment, reach, context and causal pathways [..]" Although the methods section (line 47/48 page 4) specifies "aim is to understand how CGA might be effective […]. the objectives of the process evaluation relate to implementation, mechanisms of impact and context", an specified aim/objective of the study is missing in the introduction section.<br><br><br>4. Methods and analysis: The authors wrote what they planned to do very well and extensively. But the reader may benefit from extra guidance in these paragraphs—for example, by improving the headings and more concise writing. I had to read some parts multiple times to understand what was already done (in developing this process evaluation) and what was still to be conducted (i.e. data collection and data analysis for the process evaluation itself). Herewith some of the issues I came across:<br>a. Page 4, lines 58-59; the objective may need to be written more clearly.<br>b. How will the authors use the four iterative stages? In case they refer to the headings in the methods section, I recommend using the exact same words (i.e. 'development of program theory', generation of research questions' etc, rather than the active forms

	used in the current headings).<br>c. Page 5, line 49; the authors refer to pre-specified hypotheses. Are these already specified, or is that to be done, or are the authors referring to the text on page 6 & Table 1?<br>d. Page 5; The investigators of the process evaluation are part of the primary GOAL trial (line 54/55). How could feedback or leanings be derived without informing this main trial (written in line 38/39)?<br>e. Do I understand correctly that the literature review resulted in the logic model (Figure 2) that led to the program theory (text below figure 2), which in turn directed the research questions as presented in Table 1? Or are these still subject to evolvement during the process evaluation (as suggested for the program theory on page 12, line 34)?<br>f. Page 6 lines 30-45: this paragraph may benefit readability by shortening extensive sentences.<br>g. Table 1: Are the instruments/components used in the CGA, presence of disciplines in the MDT Meetings, presence of CGA outcome discussions with patients, and management support, also considered relevant components for data collection for this process evaluation?<br>h. Table 1: Please note that an abbreviation was not spelled out (BCT).<br>i. Page 10, line 22-27: What information is extracted from the redacted CGA letters?<br>j. Page 10, line 40: will the interviews with health professionals and patients be done separately?<br>k. Page 11, line 28: There was also an interview guide for administrative staff in the appendices. Would the authors like to mention these persons here as well?<br>l. Are nephrology nurses or geriatric nurses involved in the CGA? No interview guide was appointed for these professions (in supplementals).<br>m. It is stated multiple times that informed consent will be signed by participants, which is redundant.<br><br>5. There is a lot of detail on data generation (pages 9-11). The authors refer to the COREQ checklist for reporting qualitative research. It seems to me that the authors have used the COREQ guidance for reporting this protocol. The COREQ checklist is appropriate for reporting qualitative research (e.g. the interview study), but not necessary for the publication of this protocol. I would suggest the author omit some of the written text, amongst other parts e.g. details on the personal characteristics of the research team (lines 32-39, page 11).<br><br>6. Discussion: Are there any other process evaluations ongoing or done about CGA in CKD? Have you been informed by any published literature on barriers and facilitators for (modified) geriatric assessment in CKD care (e.g. by Hall et al., 2016 J Am Geriatr Soc; Brown et al., 2019 CJASN; Nixon et al., 2021 J Nephr; Voorend et al., 2021 Eur Geriatr Med; Voorend et al., 2021 BMC Nephrology)?<br><br>7. Supplements:<br>a. Have the authors considered investigating to what extent the CGA's (e.g. instruments used) differed between the sites? And what elements of the performed CGA were perceived as valuable and acceptable? (In the Interview Guide for patients, the investigators ask: "which parts of the assessment held the most value for you?"

	Why did they not include this question in the interview guides for geriatricians and nephrologists?)<br>b. Do you investigate if there are subgroups that may be of particular interest to CGA?<br>c. Interview guide for caregivers; Q2 – probably an informal caregiver will often refer to the patient as a parent. Q3 – does this question refer to a hetero-anamnesis conducted by the geriatrician, any caregiver burden assessments, or to the caregiver being an observer in the assessment done by the patient?

**REVIEWER**	Antonio Bellasi<br>Azienda Ospedaliero Universitaria di Ferrara Arcispedale Sant'Anna, Nephrology and Dialysis
**REVIEW RETURNED**	24-Feb-2024

**GENERAL COMMENTS**	Dear editor,<br>I have read the manuscript with great interest. While it is nicely crafted it is unclear the real goal of the publication other than dissemination of the study protocol. As stated on page 6 the study closed in July 2023 and the study protocol was published already (reference 20). The discussion do not clarify what this article add to what already available in the literature

**REVIEWER**	Liz Steed<br>Barts and The London School of Medicine and Dentistry, Centre for Primary Care and Public Health
**REVIEW RETURNED**	02-May-2024

**GENERAL COMMENTS**	This is a well written paper and process evaluations are important elements of understanding interventions undergoing an RCT, so are to be welcomed. Publication of protocols is to be supported to increase objectivity within the field. Although I do not have any major concerns in relation to the paper there are elements which I feel would strengthen the paper:-<br><br>The authors report that this is theory based process evaluation and it is good to see that there is a logic model and programme theory. It is not clear however if any mid range theories were considered either for change in the intervention (I note there are mention of BCTs but not underpinning theory related to these) or indeed implementation. Were any frameworks such as CFIR considered. If these were not included a note to recognise why and the potential limitation of this in the discussion would be important.<br><br>The authors note in their summary points that they did not measure fidelity of intervention delivery. I feel this, and the limitation it presents should be picked up in the discussion.<br><br>Although there is a comment about what CGA is at the end of the first paragraph I feel this could be expanded to give the reader a better understanding of what CGA looks like in real life e.g. in particular what sort of integrated care plan - who delivers these and then reviews<br><br>Although it is good that the main data collector for the process evaluation is independent the two other researchers on this part of the project are investigators on the main trial. This is realistic given resources but this limitation (or indeed some of its strengths) could be reflected upon in discussion

	Sampling - was consideration given to sample individuals who did/did not engage in the intervention. It is good to see that some HCPs are interviewed but are these people conducting/supporting goal setting. Are any other members of the MDT included? Please clarify.<br><br>I am not clear if the patient-care giver interviews are dyadic (although topic guide doesn't imply this). Is the target therefore 64 patients and 64 caregivers - this is huge numbers can the authors clarify if this is the case.<br><br>I wonder if the section on semi-structured interviews could be made concise - there is quite a lot of repetition between sections.<br><br>The first paragraph of data analysis section is not very clear. It discusses that if themes emerge they will be considered qualitatively but unless qualitative analysis is being done how will themes emerge - may just want to clarify this paragraph. It is noted a deductive approach to coding will be used - what will guide this?<br><br>The discussion should reflect on the limitations of the study as noted in comments above.

VERSION 1 – AUTHOR RESPONSE

A. **[Choose date]Reviewer 1: Dr. Asad Merchant, University of Toronto**

Reviewer Comment	Author Response	*Revised Text* (Location in Marked Copy of revised manuscript)
**1. The protocol paper might benefit from a more detailed description of the qualitative research methodological theory that is to be used to analyse the data, including an explanation of program theory.**	Development of program theory is the first stage of the process evaluation work. This is noted in the original text, on page 6, lines 6-7 of the revised manuscript.<br><br>We agree that mention of the underlying epistemological approach is important. As such, we have adapted the Abstract and Main Document.	*"…utilising a reflexive thematic analysis, with both inductive and deductive approaches underpinned by an interpretivist perspective."* (Page 1, Lines 27-28)<br><br>*"Qualitative data, from CGA letters, process data and semi-structured interviews will be analysed using a Reflexive Thematic Analysis, underpinned by an interpretivist epistemological position, using both deductive and inductive approaches."* (Page 13, Lines 27-30)
**2. Recruitment of patients is not clear - there do not appear to be specific**	The inclusion criteria for the main outcome trial are included in the GOAL trial	*"All patients and caregivers who participate in the main trial will be eligible to interview. An effort*

**inclusion criteria. Frailty index and age and co-morbidities are listed as characteristics rather than clear inclusion criteria.**	main protocol. These include adults age greater than or equal to 65 years (or greater than or equal to 55 years for Aboriginal and Torres Strait Islander patients) with chronic kidney disease stages 3-5 (including dialysis) with a Frailty Index >0.25[1] All patients who participate in the GOAL trial will be offered the chance to participate in the process evaluation, with researchers making effort to capture a wide range of different stakeholders including those from different geographical locations and with different health status including stages of chronic kidney disease and frailty. We have edited this section to improve clarity.	*will be made to capture a broad range of patients, with varying degrees of age (including those >85 years), frailty (including those with Frailty Index >0.4) and medical comorbidities." (Page 11, Lines 26-29)*
B. **Reviewer 2: Dr. Carlijn Voorend, Leiden University Medical Center**		
**Reviewer Comment**	**Author Response**	***Revised Text* (Location in Marked Copy of revised manuscript)**
**1. Fox and colleagues described the protocol of a study that aims to understand how CGA might be effective for older frail adults with CKD in the outpatient setting. They planned to conduct a process evaluation of the cluster randomised controlled GOAL trial (protocol published by Logan et al., 2023 in Trials). They specifically aim to investigate implementation, mechanisms of impact and context of the CGA intervention, which are the three key components of the MRC guidance for process evaluation of complex interventions. Amongst**	Thank you for these comments.	NA

**other resources (implementation and recruitment data, medical letters), the researchers aim to survey PIs and research coordinators, and conduct additional interviews with research coordinators, research staff, and administrative staff. I applaud the authors' plans to conduct an extensive structured process evaluation for the complex CGA intervention in CKD care. This study will contribute valuable information for integrating geriatric and nephrological care. My questions and remarks to consider for publishing the protocol are in the attached file.**		
2. **The title is precise and informative, though long. The authors may want to consider omitting 'a pragmatic cluster randomised controlled trial' since this concerns the study design of the GOAL trial rather than the current process evaluation. They could also consider changing 'whether' for 'how', fitting the aim of the current evaluation study.**	We have modified the title.	*"A Protocol for the Process Evaluation of the GOAL Trial: investigating how comprehensive geriatric assessment (CGA) improves patient-centred goal attainment in older adults with chronic kidney disease in the outpatient setting*".(Page 1, Title)
3. **(Abstract) The investigators may want to consider including a sentence on how outcomes of this process evaluation are assumed to benefit care/research.**	We have added a sentence to the abstract.	*"This process evaluation will offer novel insights into how and why CGA might be beneficial for frail older adults with CKD, and provide guidance when considering how to implement this complex intervention into clinical practice*." (Page 1, Lines 15-17)
4. **(Abstract) : Is it common for BMJ Open to include**	We have removed the references from the abstract.	(Page 1, Line 20)

**references in the abstract?**		
5. **(Abstract) Reviewer comment: Have you considered if the STARI checklist or SQUIRE guideline would fit the purpose of reporting the outcomes of the process evaluation?**	*We do not view this primarily as an implementation study, and therefore do not think the STARI checklist is best suited. However, we agree that referral to the SQUIRE guidelines will be valuable when reporting the outcomes of our process evaluation. We have amended the text accordingly.*	*"The SQUIRE guidelines will also be considered when reporting the results of the process evaluation."* (Page 1, Lines 30-31)<br><br>*"The SQUIRE guidelines will be considered when reporting the results of the process evaluation.[(2)]"* (Page 13, Lines 42-47)
6. **(Introduction) (page 4, lines 25-35): Could you provide some sentences to inform the reader about the intervention of the GOAL Trial? (e.g. what CGA does it exist of, by whom is it conducted, where and how?)**	We have added to the text.	*"The pragmatic design of the trial means that geriatricians are asked to provide CGA as they usually would in their clinical practice. This is a comprehensive, multidimensional assessment provided by a geriatrician in the outpatient setting. No proforma or structure to the CGA is mandated, and it is at the discretion of the geriatrician to what degree they employ multidisciplinary input or ongoing follow-up." (Page 4, Lines 21-26)*
7. **(Introduction) In the GOAL-trial protocol, Logan et al describe "a process evaluation will be conducted [..] whether the intervention was delivered as intended, any issue or local barriers to invention delivery, and perceptions of the intervention by participants." In the introduction (final sentence) of the current manuscript the authors state: "we will be conducting a dedicated process evaluation of**	This was an unintentional omission. We have now included implementation in the introduction.	*"Upon completion of trial recruitment and follow-up, we will be conducting a dedicated process evaluation of the GOAL Trial, focusing on implementation, recruitment, reach, context and causal pathways, as described in this protocol."* (Page 4, Line 31)

	**the GOAL trial, focusing on recruitment, reach, context and causal pathways [..]" Although the methods section (line 47/48 page 4) specifies "aim is to understand how CGA might be effective […]. the objectives of the process evaluation relate to implementation, mechanisms of impact and context", an specified aim/objective of the study is missing in the introduction section.**		
8.	**(Methods) The authors wrote what they planned to do very well and extensively. But the reader may benefit from extra guidance in these paragraphs—for example, by improving the headings and more concise writing. I had to read some parts multiple times to understand what was already done (in developing this process evaluation) and what was still to be conducted (i.e. data collection and data analysis for the process evaluation itself). Herewith some of the issues I came across:**	We have reviewed the structure and headings to improve readability.	NA
**9.**	**(Methods) Page 4, lines 58-59; the objective may need to be written more clearly.**	We have edited this section considerably with an aim to be more concise.	*"A key objective is an exploration of how CGA was implemented in the trial context, including recruitment processes (who was recruited and how), and how the intervention was delivered to patients. Other key objectives include exploration of patient response to the intervention and perceived*

		acceptability to patients, unexpected consequences of the intervention, and broader contextual factors that influenced implementation and patient experience. The aim is that this exploratory analysis will allow further refinement of program theory, to understand the impact of contextual factors on causal mechanisms."(Page 5, Lines 14-20)<br><br>(Deleted text): ~~*"This includes investigating how clusters were recruited, recruitment of patients, response of clusters and individuals, delivery to patients, unexpected consequences, program theory and broader context. We have also been guided by previously published process evaluations of complex healthcare interventions.*~~[16, 26, 27]<br><br>~~*The objectives of the process evaluation relate to implementation, mechanisms of impact and context.*~~<br><br>~~*Implementation considerations include*~~<br><br>~~*Recruitment processes of sites*~~<br>~~*Recruitment processes of individuals*~~<br>~~*Reach and characteristics of individuals, and*~~<br>~~*Processes relating to delivery of the intervention to individuals.*~~<br>~~*Mechanisms of impact involves questioning:*~~<br><br>~~*How CGA might work to improve attainment of patients' own goals in the outpatient setting (program theory)*~~<br>~~*The components of CGA that make it effective for patients*~~

		~~*with CKD to achieve their goals in the outpatient setting*~~<br>~~*For what groups of people and in what circumstances CGA provides benefit*~~<br>~~*The acceptability of CGA to patients in the trial context, and how their perception and responses to the intervention influences causal pathways*~~<br>~~*How CGA is perceived and used by professional stakeholders and how this interacts with implementation and outcomes*~~<br>~~*Unexpected consequences of CGA not captured in the main trial outcomes data*~~<br>~~*Broader Context refers to an understanding of:*~~<br><br>~~*How characteristics of the intervention interact with context (defined as the setting in which the CGA is delivered, including the hospital, health professionals and interactions between the patients and the health care service) to augment outcomes*~~<br>~~*Contextual barriers and enablers which impact intervention implementation and outcomes."*~~<br>(Page 5, Line 20 – Page 6, Line 2)
10. **(Methods) How will the authors use the four iterative stages? In case they refer to the headings in the methods section, I recommend using the exact same words (i.e. 'development of program theory', generation of research questions' etc, rather than the active forms used in the current headings).**	We modified these headings to use the same words used in the "Broad Aims and Design" section.	*"Development of program theory"* (Page 6, Line 46)<br><br>*"Generation of research questions"* (Page 7, Line 29)<br><br>*"Generation of data"* (Page 10, Line 3)<br><br>*"Analysis of Data"* (Page 13, Line 8)

11. **(Methods) Page 5, line 49; the authors refer to pre-specified hypotheses. Are these already specified, or is that to be done, or are the authors referring to the text on page 6 & Table 1?**	We have amended this section to clarify that it is part of the process evaluation process and is not yet complete.	(Please see the modified language on Page 6 Line 26 through to Page 7 Line 27, in particular as below.)<br><br>*“To date, an early program theory has been developed, which will be further developed.”* (Page 7, line 14)<br><br>*“An early version of this logic model is presented”* (Page 7, Line 9)<br><br>*“The process evaluation will allow for both deductive and inductive approaches, in an iterative process incorporating new learning through ongoing data collection, review and reflection.”* (Page 6, Lines 27-29)
**12. (Methods) Page 5; The investigators of the process evaluation are part of the primary GOAL trial (line 54/55). How could feedback or leanings be derived without informing this main trial (written in line 38/39)?**	Whilst the planning for the process evaluation incorporates all authors, analysis of process data that occur while the trial is ongoing (e.g. analysis of recruitment data) is only undertaken by the main researcher for the process evaluation (first author), who is not involved in the main GOAL trial and who will not feedback any learnings/insights to the other trial investigators. Collection of ‘new’ data (ie. Data generated specifically for the process evaluation such as the semi-structured interviews) only began after	*“The process evaluation includes key investigators involved in the main GOAL trial. However, analysis of process data is only undertaken by one researcher, the first author, who is not involved in the main trial and will not feedback results while the trial is ongoing. Furthermore, collection of data specific to the process evaluation, such as semi-structured interviews, will only commence once each site has completed last-patient, last-visit to limit the process evaluation work influencing the main trial outcomes.”* (Page 6, Lines 8-14)

	each site had finished last patient last visit at that site. The Trial Steering Committee determined that this was important so that the results of the process evaluation did not inject bias into the trial or modify the intervention. However, it was also felt that it was important that the process evaluation was integral enough to the main trial, that it could derive meaningful results and insights, thus the involvement of key trial staff in planning and managing the process evaluation work.	
13. **(Methods) Do I understand correctly that the literature review resulted in the logic model (Figure 2) that led to the program theory (text below figure 2), which in turn directed the research questions as presented in Table 1? Or are these still subject to evolvement during the process evaluation (as suggested for the program theory on page 12, line 34)?**	This sequence you describe is correct. However, this will still be subject to evolvement during the trial, with refinement and development of this preliminary program theory. We clarified this in the manuscript.	*"To date, an early program theory has been developed, which will be further developed."* (Page 7, Line 14)
14. **(Methods) Page 6 lines 30-45: this paragraph may benefit readability by shortening extensive sentences.**	We have amended this paragraph as suggested.	*"To date, an early program theory has been developed, which will be further developed. We propose that CGA works through an iterative process of assessment that is holistic and takes account of the 'whole person'. It includes development of a management plan, and care coordination, both of which require a person-centred approach, interdisciplinary working and a focus on mutually agreed goals of care. CGA works when goals that are personally meaningful to the*

		patient are framed so that a coordinated multidisciplinary management plan, that is acceptable to the patient, can be organised. The multidisciplinary team, having clear roles and effective working relationships, work with the patient to achieve goals. This means the patient and caregiver perceive value in the intervention and trust the assessments of the multidisciplinary providers. Contextual inputs, such as access to support from executive, adequate funding, physical resources and knowledge acquisition mean that treatment plans can be executed with effectiveness. Communication, inter-professionally and between the patient and health professionals, is clear and effective meaning that all members are 'on the same page'." (Page 7, Lines 14-27)
15. **Table 1: Are the instruments/components used in the CGA, presence of disciplines in the MDT Meetings, presence of CGA outcome discussions with patients, and management support, also considered relevant components for data collection for this process evaluation?**	Yes, very much so. They are included in the original Table 1 under *"Delivery to individuals: what was delivered to the individual as the CGA intervention?"*	NA
16. **(Methods) Table 1: Please note that an abbreviation was not spelled out (BCT).**	We have now corrected this.	*"BCT (Behaviour Change Technique)"* (Page 8, Table Footer/Legend)
17. **(Methods) Page 10, line 22-27: What information is extracted from the redacted CGA letters?**	We have included this information in the manuscript.	*"They will be analysed descriptively, using manual content analysis to extract the key domains assessed, multidisciplinary involvement*

		and other key components such as goal-setting and follow-up. They will also be analysed qualitatively to explore how geriatricians described the identified issues, patient goals and patient concerns, using a reflexive thematic analysis.[3, 4]" (Page 11, Lines 13-18)
**18. (Methods) Page 10, line 40: will the interviews with health professionals and patients be done separately?**	Yes, they will be done separately. We have clarified this.	"Interviewers of health professionals and patients will be conducted separately." (Page 11, Lines 31-32)
**19. (Methods) Page 11, line 28: There was also an interview guide for administrative staff in the appendices. Would the authors like to mention these persons here as well?**	We have corrected this.	*"We will attempt to include a mix of geriatricians, nephrologists, research coordinators, trial investigators and administrative staff."* (Page 11, Lines 45-46)
**20. Are nephrology nurses or geriatric nurses involved in the CGA? No interview guide was appointed for these professions (in supplementals).**	In the GOAL trial, the geriatrician is the only health professional conducting the CGA.	NA
**21. (Methods) It is stated multiple times that informed consent will be signed by participants, which is redundant.**	We have amended the relevant sections.	Deletion: *~~"Caregivers will also require patient consent to participate. Health professionals will be approached via telephone at the end of the trial period, to seek their consent for interviews."~~ (Page 12, Lines 18-20))*<br><br>Deletion: *~~"all patients and caregivers. This will occur face-to-face at the time of recruitment."~~ (Page 12, Lines 16-17))*

		Deletion: *"Informed consent to participate will be obtained from all participants."* (Page 14, Lines 13-14)
**22. (Methods) There is a lot of detail on data generation (pages 9-11). The authors refer to the COREQ checklist for reporting qualitative research. It seems to me that the authors have used the COREQ guidance for reporting this protocol. The COREQ checklist is appropriate for reporting qualitative research (e.g. the interview study), but not necessary for the publication of this protocol. I would suggest the author omit some of the written text, amongst other parts e.g. details on the personal characteristics of the research team (lines 32-39, page 11).**	We have deleted the information about the research team. We think it is valuable to mention that we will report qualitative data according to COREQ guidelines but we have removed the COREQ checklist as an appendix.	Deletion: *" Research Team*<br><br>*i) Personal characteristics: It is anticipated that most or all of the interviews will be conducted by a single researcher (STF). However, other members of the research team will be involved in analysis. All members of the research team are women. STF is a geriatrician and general physician and is completing a higher degree by research involving CGA. MJ (PhD) is the director of a health services research centre and a university academic. REH (MD) is a geriatrician and clinical academic.*<br>*ii) Relationship with participants*<br>*The researchers do not have a prior relationship with most of the interview participants. The participants will be given only a brief introduction to the interviewer.*<br>*iii) Positionality and Bias: All researchers involved in qualitative analyses will formulate a positionality statement, a version of which will be*

		~~*included in the final manuscript.”*~~<br>(Page 12, Lines 31-45)<br><br>Deletion: ~~*“The COREQ checklist is included as Appendix H.”*~~<br>(Page 13, Line 6)<br><br>(N.B. The remainder of this section has moved to later in the manuscript)<br><br>*“Reporting guidelines*<br><br>*The SQUIRE guidelines will be considered when reporting the results of the process evaluation.*[32] *The qualitative components of this work, in particular the interviews, will be reported according to the Consolidated criteria for reporting qualitative research (COREQ) guidelines.*[33] *“* (Page 13, Lines 42-47)
**23. (Discussion) Are there any other process evaluations ongoing or done about CGA in CKD? Have you been informed by any published literature on barriers and facilitators for (modified) geriatric assessment in CKD care (e.g. by Hall et al., 2016 J Am Geriatr Soc; Brown et al., 2019 CJASN; Nixon et al., 2021 J Nephr; Voorend et al., 2021 Eur Geriatr Med; Voorend et al., 2021 BMC Nephrology)?**	Please note, following Editorial comment, this section has been moved to the end of the introduction. Yes, these are all excellent references that have informed our research. We agree it is helpful to mention them.	*“CGA may be particularly suited to older adults with CKD who have high degrees of frailty and functional impairment.*[5, 6] *Process evaluations have occurred for implementation of specific CGA programs in nephrology care. These have suggested CGA can help anticipate and manage risks, identify problems and focus decision-making to be patient-centred.*[7, 8]*However, a challenge for CGA in this cohort is ensuring that goals and assessments are communicated appropriately to patients. Effective multidisciplinary working has been identified as important in ensuring CGA is beneficial for this group.*[9]*”*<br>(Page 4, Lines 10-16)

**24. (Supplements) Have the authors considered investigating to what extent the CGA's (e.g. instruments used) differed between the sites? And what elements of the performed CGA were perceived as valuable and acceptable? (In the Interview Guide for patients, the investigators ask: "which parts of the assessment held the most value for you?" Why did they not include this question in the interview guides for geriatricians and nephrologists?)**	We will not be looking specifically at inter-site variation, nor measuring this difference in a statistical way. However, we will be collecting data on the breadth of ways in which CGA is used (e.g. which components are used variously across sites) and we will also be asking patients, caregivers and health professionals what they perceive to be valuable in a CGA. We have included this in the original interview guides for geriatricians, and in a modified way for the nephrologists (who are not present for the CGA).	(Unedited, from original):<br><br>*"Based on your experiences with this study, do you think it would be good to have a geriatrician integrated into the care team for older frail patients with chronic kidney disease? If yes, why; if no, why not? Prompts: Can you describe to me what it is about the CGA intervention, including what happened during the consultation and what the follow-up /review, that makes it beneficial or not?"* (Appendix D, Dot Point 5)<br><br>(Unedited, from original):<br><br>*"CGA Acceptability and Value Based on your experiences with this study, do you think it would be good to have a geriatrician integrated into the care team for older frail patients with chronic kidney disease? If yes, why; if no, why not? Prompts: Positive things: benefits of in-depth assessment of goals of patients; good to share the care for frail patients Negative things: decision making can be done with another health care professional already; patients feel too unwell or overwhelmed at the time; kidney team is taking good care of the patients; patients already have multiple providers - adding the geriatrician makes it more complex; geriatrician comes and goes but we must then action the prescriptions/plans, that is the hard part; geriatricians explanations come too late; patients already set their mind on a path forward etc.) Did you speak to others about the*

		intervention, and would you recommend other teams to integrate a comprehensive geriatric assessment into their care pathways?" (Appendix E, Dot Point 7
**25. (Supplements) Do you investigate if there are subgroups that may be of particular interest to CGA?**	A subgroup analysis will not be possible during the process evaluation. However, themes may emerge about particular groups that benefit through the qualitative analysis The main trial outcomes paper subgroup analysis will be able to differentiate effect size based on degree of frailty, age, comorbid status etc.	
**26. Interview guide for caregivers; Q2 – probably an informal caregiver will often refer to the patient as a parent. Q3 – does this question refer to a hetero-anamnesis conducted by the geriatrician, any caregiver burden assessments, or to the caregiver being an observer in the assessment done by the patient?**	Re Question 2: We have updated the interview guide<br><br>Re Question 3: This refers to the caregiver's recollection and impression of the consultation between the geriatrician and patient, at which they were present. (Appendix G, Dot Points 2 and 3)	*"Are you able to describe how you came to be involved in the GOAL study and what your thoughts were about your (friend/partner/parent etc) being involved?"*<br><br>(Appendix G, Page 1, Line 23-24)
C. **Reviewer: 3 Dr. Antonio Bellasi, Azienda Ospedaliero Universitaria di Ferrara Arcispedale Sant'Anna**		
**Reviewer Comment**	**Author Response**	***Revised Text*** **(Location in Marked Copy of revised manuscript)**
**1. I have read the manuscript with great interest. While it is nicely crafted it is unclear the real goal of the publication other**	While the study protocol for the main GOAL outcomes trial has been published, this only made brief mention of the process evaluation. Process evaluations add	NA

**than dissemination of the study protocol. As stated on page 6 the study closed in July 2023 and the study protocol was published already (reference 20). The discussion do not clarify what this article add to what already available in the literature.**	significant value when interpreting trials of complex healthcare interventions, ensuring results can be interpreted in a way that takes account of context and allows implementation into policy and practice. To ensure trustworthiness of process evaluations, methodology must be clear and transparent. This allows clearer interpretation of results, and also creates opportunity for others to build on this work in other process evaluations and implementation studies. Publishing detailed protocols for process evaluations of complex healthcare interventions is an important contribution to the literature.{French, 2020 #76}	
D. **Reviewer 4 Dr. Liz Steed, Barts and The London School of Medicine and Dentistry**		
**Reviewer Comment**	**Author Response**	***Revised Text*** **(Location in Marked Copy of revised manuscript)**
**1. This is a well written paper and process evaluations are important elements of understanding interventions undergoing an RCT, so are to be welcomed. Publication of protocols is to be supported to increase objectivity within the field. Although I do not have any major concerns in relation to the paper there are elements which I feel would strengthen the paper:-**	Thank you for these comments.	NA

**2. The authors report that this is theory based process evaluation and it is good to see that there is a logic model and programme theory. It is not clear however if any mid range theories were considered either for change in the intervention (I note there are mention of BCTs but not underpinning theory related to these) or indeed implementation. Were any frameworks such as CFIR considered. If these were not included a note to recognise why and the potential limitation of this in the discussion would be important.**	*Because this was not primarily an implementation study, and most of the process evaluation work followed the main outcomes trial, we did not feel that the CFIR was most appropriate. Our approach primarily follows the MRC guidance for process evaluations of complex healthcare interventions. We feel it is outside of the scope of the process evaluation to take an approach centred primarily on mid-range theories such as BCT, however, there may be scope to explore these as secondary analyses/studies after the main process evaluation. We agree that it is important to discuss both of these points in the discussion. As per Editorial advice, there is no discussion in this protocol paper. Therefore, we will endeavour to include it when presenting the results of the process evaluation.*	NA
**3. The authors note in their summary points that they did not measure fidelity of intervention delivery. I feel this, and the limitation it presents should be picked up in the discussion.**	Assessing fidelity is an important component of the process evaluation. What this Summary point is trying to say is that the process evaluation is occurring after, and separate to, the main trial. Therefore, learnings from the process evaluation, including about fidelity, cannot be fed back to 'improve' the trial intervention. To clarify this, we have deleted from this Summary point the part that says ', including barriers, enablers or intervention fidelity'.	(Deletion):~~*"…including barriers, enablers or intervention fidelity."*~~ (Page 2, Line 8)
**4. Although there is a**	The CGA delivered in the trial was at the discretion of	*"The pragmatic design of the trial means that geriatricians are*

**comment about what CGA is at the end of the first paragraph I feel this could be expanded to give the reader a better understanding of what CGA looks like in real life e.g. in particular what sort of integrated care plan - who delivers these and then reviews.**	the geriatrician. We have included some extra text to clarify this.	*asked to provide CGA as they usually would in their clinical practice. This is a comprehensive, multidimensional assessment provided by a geriatrician in the outpatient setting. No proforma or structure to the CGA is mandated, and it is at the discretion of the geriatrician to what degree they employ multidisciplinary input or ongoing follow-up.”*<br><br>(Page 4, Line 21-26)
**5. Although it is good that the main data collector for the process evaluation is independent the two other researchers on this part of the project are investigators on the main trial. This is realistic given resources but this limitation (or indeed some of its strengths) could be reflected upon in discussion.**	We agree that this is an important point of discussion. As there is no discussion in protocol papers, as per Editorial direction, we will discuss this with the results of the process evaluation when available.	NA
***6.* Sampling - was consideration given to sample individuals who did/did not engage in the intervention. It is good to see that some HCPs are interviewed but are these people conducting/supporting goal setting. Are any other members of the MDT included? Please clarify.**	We will focus primarily on patients who received the intervention but will also interview a small number of patients and caregivers from control sites. We have amended the manuscript to improve clarity.<br><br>With regards to healthcare professionals, research coordinators will be interviewed, who will have conducted the goal setting. No other members of the MDT will be actively included as the trial coordinating centre does not have details of their names or roles at	*“We will aim for at least four patient and one caregiver interview per intervention site, two patient or caregiver interview from each control site, and a total of 20 health professional interviews, continuing until thematic saturation is achieved”.* (Page 11 Line 47 – Page 12, Line 3).<br><br>*“If available, we will also interview multidisciplinary health professionals.”* (Page 11 Lines 46-47)

	each site. Research coordinators will be asked to provide names of all people involved in delivering the GOAL trial at each site, and if this includes MDT we will invite them to be interviewed. We have included a line to clarify this also.	
**7. I am not clear if the patient-care giver interviews are dyadic (although topic guide doesn't imply this). Is the target therefore 64 patients and 64 caregivers - this is huge numbers can the authors clarify if this is the case.**	There is the option for dyadic or individual interviews of caregivers. We have clarified this in the manuscript.<br><br>The way we expressed the numbers may have been unintentionally misleading. We have clarified this in the manuscript. A total of approximately 60-65 interviews is the aim.	*"Interviews of caregivers can be conducted individually or dyadically*." (Page 12, Lines 27-28)<br><br>*"We will aim for at least four patient and one caregiver interview per intervention site, two patient or caregiver interview from each control site, and a total of 20 health professional interviews, continuing until thematic saturation is achieved."* (Page 11, Line 47 – Page 12, Line 3)<br><br>*"Approximately 60 semi-structured interviews will be analysed with a qualitative approach…"* (Page 1, Line 26)
**8. I wonder if the section on semi-structured interviews could be made concise - there is quite a lot of repetition between sections**	*We have shortened and revised this section. We have deleted the first paragraph in this section, as this includes a lot of detail that is repeated in the dot points a) through e). We have also tried to delete other less important information from this section.*	(Deleted): *~~"At the completion of follow-up for the intervention period for each site (last patient, last visit), semi-structured interviews will be carried out with purposively sampled patients. If caregivers are available and provide consent, some interviews will also be carried out with caregivers to seek their opinions in addition to those of patients. Care will be taken to capture maximum clinical and demographic~~*

		diversity. Health professionals will also be approached to participate in semi-structured interviews." (Page 11, Lines 23-28)<br><br>(Deleted): "Research Team Personal characteristics: It is anticipated that most or all of the interviews will be conducted by a single researcher (STF). However, other members of the research team will be involved in analysis. All members of the research team are women. STF is a geriatrician and general physician and is completing a higher degree by research involving CGA. MJ (PhD) is the director of a health services research centre and a university academic. REH (MD) is a geriatrician and clinical academic.<br>Relationship with participants The researchers do not have a prior relationship with most of the interview participants. The participants will be given only a brief introduction to the interviewer.<br><br>Positionality and Bias: All researchers involved in qualitative analyses will formulate a positionality statement, a version of which will be included in the final manuscript."<br>(Page 12, Lines 31-45)<br><br>(Current Text):<br><br>*"Sampling and Sample Size: We will purposively sample health professionals, patients and caregivers for semi-structured interviews. Interviewers of health professionals and patients will be conducted*

		~~*separately. All patients and caregivers who participate in the main trial will be eligible to interview. An effort with be made to capture a broad range of patients, with varying degrees of age (including those >85 years) , frailty (including those with Frailty Index >0.4) and medical comorbidities. [30, 31] Of note, the GOAL trial protocol excludes patients with significant cognitive impairment who cannot consent for themselves. However, trial patients with mild cognitive impairment will be included. Given that we will rely on approached patients to consent to the interviews, convenience sampling will also be necessary, in addition to the purposive sampling described above. We will attempt to include a mix of geriatricians, nephrologists, research coordinators, trial investigators and administrative staff. If available, we will also interview multidisciplinary health professionals. We will aim for at least four patient and one caregiver interview per intervention site, two patient or caregiver interview from each control site, and a total of 20 health professional interviews, continuing until thematic saturation is achieved. Recruitment: Recruitment for the patient and caregiver interviews will commence when each site has completed all follow-up for all patients (that is when the last patient at that site has completed their 12-month follow-up). Recruitment for health professionals will occur when all sites have completed all follow-up. This decision was made to prevent contamination and bias from the process evaluation affecting the main*~~

		~~*trial outcomes.*~~<br>~~*Consent for interviews: Written consent will be obtained for all participants interviewed Consent for the interviews is not necessary for patient participation in the main trial.*~~<br>~~*Interview Processes: Interviews will be conducted via audio-visual communication or telephone, and will be audio-recorded using a digital recording device. Field notes may also be taken. Interview questions will be open-ended, and phrased to encourage patients to discuss their own experiences and opinions. Medical and research jargon will be avoided. No repeat interviews will be carried out. Each interview will take approximately 30-60 minutes. Transcripts will not be returned to participants. No non-participants will be present for the interview. Interviews of caregivers can be conducted individually or dyadically.*~~<br>~~*Interview Guides: Copies of the interview guides for research coordinators, geriatricians, nephrologists, patients, and caregivers are available as Appendices C-G."*~~<br>(Page 11, Line 30 – Page 12, Line 30)
**9. The first paragraph of data analysis section is not very clear. It discusses that if themes emerge they will be considered qualitatively but unless qualitative analysis is being done how will themes emerge - may just want to clarify this paragraph. It is noted a deductive approach to coding will be used - what will guide**	We have modified this section significantly, to make it clearer. The revised text is provided in the adjacent column.	*"All implementation data (issues register, recruitment emails, trial site meetings) will be logged into spreadsheets. Descriptive statistics will be used to summarise issues noted in the process data (issues register as well as recruitment discussions), and descriptive data in the CGA letters. Quantitative data about the interview participants will also be described descriptively.*

**this?**		*The interviews will be recorded and transcribed verbatim. Pseudonyms will be used in place of patient names and as such the stored information will be in a de-identified format. All identifiable data will be stored on password-protected files. The signed consent forms for patients and caregivers will be stored by the central coordinating centre, while those for the health professionals will be stored by a member of the process evaluation team.*<br><br>*Qualitative data, from CGA letters, process data and semi-structured interviews will be analysed using a Reflexive Thematic Analysis, underpinned by an interpretivist epistemological position, using both deductive and inductive approaches.* [29, 35] *The deductive component will be informed by the program theory and logic model developed in the first stage of the process evaluation. STF and one other team member, will complete data coding. A copy of the coding tree will be provided with the published results. These will then be synthesised to further develop the program theory that takes account of potential causal mechanisms Participants will be invited to provide feedback on the findings. All qualitative data will be analysed with NVivo software, and presented in a narrative format with excerpts of interviews included to illustrate key points.* [34] *"*<br><br>(Page 13, Lines 4-34)

**10. The discussion should reflect on the limitations of the study as noted in comments above.**	We agree this is important. As there is not scope to include a discussion in the protocol paper, we will ensure this is included when the results are reported.	

VERSION 2 – REVIEW

**REVIEWER**	Asad Merchant<br>University of Toronto
**REVIEW RETURNED**	28-May-2024

**GENERAL COMMENTS**	Comments BMJ Open –<br>Methods and Analysis<br>Line 4 – “The aim of the process evaluation is to understand how CGA might be effective for older frail adults with CKD in the outpatient setting” – Effective in what way? What are the outcomes we are hoping to achieve? Whether CGA can be effectively administered in clinic, or if it makes a difference in care?

**REVIEWER**	Carlijn Voorend<br>Leiden University Medical Center
**REVIEW RETURNED**	23-Jun-2024

**GENERAL COMMENTS**	The authors have substantially improved their manuscript. I have some minor remarks and suggestions.<br>- Introduction page 4, line 32-33: The authors state that de GOAL trial is still ongoing and expected to close by July 2023. (almost a year ago) Could they please explain this?<br>- Figure 3 shows numbers between brackets, where does these numbers refer to? First I assumed these should be references, but content-wise it does not seem to match?

**REVIEWER**	Liz Steed<br>Barts and The London School of Medicine and Dentistry, Centre for Primary Care and Public Health
**REVIEW RETURNED**	12-Jun-2024

**GENERAL COMMENTS**	I think this paper now reads well. I have a couple of very minor points.<br><br>i) on pg 5 please check your referencing. You say 'Our approach incorporates the MRC guidance for process evaluations of complex health care interventions, updated in 2021(Skivington) from previous guidance published in 2008(Craig) and 2015(Moore)'<br><br>I think Skivington is an update of the design and evaluation of complex interventions from Craig, whilst only Moore is guidance for process evaluations (although of course the two relate) and I'm not aware this has been updated.<br><br>ii) on page 5 ln 28 you say the analysis is only done by one

	researcher, however below you say it is done and then discussed with other authors. In reality appropriate qual analysis will always include the team as you are doing. I think perhaps just rephrase the above line to say something like the initial analysis will be conducted by one independent researcher.
